# Human-centred design of digital health dashboards in care of older adults: a scoping review

**DOI:** 10.1136/bmjopen-2025-113525

**Published:** 2026-07-17

**Authors:** Syeda Taqya Amna Arslan, Siobhan O’Connor, Bibhusha Karki, Chloe French, Emma Stanmore

**Affiliations:** 1 School of Health Sciences, The University of Manchester, Manchester, UK; 2 Florence Nightingale Faculty of Nursing, Midwifery and Palliative Care, King’s College London, London, UK; 3 School of Health Sciences and Manchester Academic Health Science Centre (MAHSC), University of Manchester, Manchester, UK; 4 Manchester University NHS Foundation Trust, Manchester, UK

**Keywords:** Aging, Decision Making, Delivery of Health Care, Integrated, Digital Technology, eHealth, Health Services for the Aged

## Abstract

**Abstract:**

**Objective:**

This review explores the conceptual frameworks, methods, barriers and facilitators associated with the human-centred design (HCD) process employed when developing digital health dashboards (DHDs) for the care of older adults.

**Design:**

This scoping review is designed according to the Preferred Reporting Items for Systematic Reviews and Meta-Analyses Extension for Scoping Review (PRISMA-ScR) guidelines, ensuring a systematic approach to identifying and synthesising relevant literature.

**Data sources:**

Five databases were searched including EMBASE, MEDLINE, PsychInfo, CINAHL and Cochrane Library from January 2012 to 30 March 2026.

**Eligibility criteria:**

Studies were eligible if they involved the HCD of DHDs for health management in older adults aged 60 years and over. The design process could involve a range of stakeholders, including older adults, healthcare professionals, carers, technology experts and other relevant parties involved in dashboard design.

**Data extraction and synthesis:**

Search terms included older persons, ageing, decision support systems, dashboards, human-centred design etc. Study screening was done in Rayyan, and data extraction was conducted in MS Excel using the Joanna Briggs Institute data extraction tool. HCD approaches were mapped to the Double Diamond framework using thematic analysis.

**Results:**

A total of 15 studies were included which employed iterative HCD approaches, involving stakeholders at various stages of dashboard development. Several conceptual frameworks were identified including user-centred design and behaviour change frameworks as well as HCD methods identified including user interviews, focus groups, co-design workshops, usability testing and surveys. Key facilitators included ongoing user engagement, multidisciplinary collaboration and iterative prototyping. Barriers included limited digital literacy among older adults, challenges in recruiting diverse user groups and resource constraints impacting the breadth of HCD activities.

**Conclusion:**

Dashboards developed using HCD approaches suggest improved usability, acceptability and relevance for older adults, supporting better self-management of health conditions. However, inconsistent frameworks, lack of research outside Europe, America and Australia and underuse of dashboard analytics were identified as challenges. Future research should adopt validated HCD frameworks, address acceptability factors and evaluate real-world use to develop more inclusive and sustainable digital health tools for older adults.

**STRENGTHS AND LIMITATIONS OF THIS STUDY**

- Searches were conducted across five databases to maximise the identification of relevant studies on the topic.
- Titles/abstracts and full texts were screened in Rayyan by two reviewers while documenting the reasons for exclusion, and any conflicts in decision were resolved among the reviewers with a third reviewer acting as an intermediary in case of disagreement.
- Established methods, including the Joanna Briggs Institute (JBI) scoping review guidance, the JBI data extraction tool, Preferred Reporting Items for Systematic Reviews and Meta-Analyses Extension for Scoping Review (PRISMA -ScR) reporting and the PCC framework for eligibility criteria were adhered to, while the quality appraisal of studies was conducted using the Mixed Methods Appraisal Tool for mixed methods studies and the JBI checklist for qualitative studies.
- The review only includes English language articles, possibly excluding relevant studies through language bias.
- Dashboards used in routine care that remain unpublished, including those developed in collaboration with stakeholders, were not systematically captured, potentially leading to an under-representation of real-world implementations.

## Introduction

 The WHO predicts one in six people will be 60 years or over by 2030, leading to a higher incidence of chronic health conditions.^1^ This demographic shift creates a need to support older adults with managing their health. Digital health tools such as mobile health apps, web-based systems and digital dashboards can facilitate the self-management of health in older adults and potentially reduce the need for clinical visits and healthcare costs.^2 3^

Digital health dashboards (DHDs) defined as ‘any electronic system designed to aid directly in clinical decision making’^4^ may be helpful in presenting complex health information in health apps, web-based systems and other digital health tools. By using visual aids and accessible formats, DHDs may enhance decision-making for healthcare professionals and patients and could lead to improved health outcomes and patient engagement.^2 5^

For the purposes of this review, a dashboard is defined as any digital tool or feature that provides visualisation, decision support, or summary overviews relevant to the care of older adults, even if the term ‘dashboard’ is not explicitly used. During study selection, design context, visual elements and user interface descriptions were considered as indicative of dashboard development where appropriate.

Although DHDs are often clinician-facing, they are increasingly being designed for older adults to support self-management or enable remote monitoring of older patients by healthcare professionals. However, research suggests that many digital tools are not designed with end users in mind, resulting in usability barriers, poor personalisation and reduced adoption and long-term use.^6^ Human-Centred Design (HCD), ‘an approach to interactive systems development that aims to make systems usable and useful by focusing on users, their needs and requirements^7^’, has emerged as a useful methodology in ensuring that digital tools, including dashboards, are accessible, acceptable and relevant to older adults.

While several studies have used HCD approaches by including older adults in the creation of digital health technologies,^6^ no review has yet synthesised evidence on how HCD specifically supports development of DHDs for the care of older adults.

A recent review on the design of DHDs highlighted that only 30% of articles referenced the use of HCD in dashboard development,^8^ while primarily providing overviews of national public health dashboards. This underscores a significant gap in dashboard design for addressing the unique needs of older adults, particularly those that incorporate older adult data and support self-management. This scoping review was therefore conducted to map the available literature on HCD methodologies for developing DHDs incorporating self-reported data from older adults. The review aims to identify any conceptual frameworks used in HCD, as well as any specific methods and tools used, while also identifying any barriers or facilitators in the design process.

The primary questions guiding this scoping review are:

1. What approaches (conceptual frameworks, methods and tools) are used in HCD to create DHDs for monitoring self-management of older adults’ health in care for older adults?
2. What are the barriers and facilitators in HCD for DHDs?

## Methods

The scoping review was conducted in accordance with the Joanna Briggs Institute (JBI) methodology, on scoping reviews and the Preferred Reporting Items for Systematic Reviews and Meta-Analyses Extension for Scoping Review (PRISMA-ScR) checklist.^9^,^12^ (online supplemental appendix I). The review has been registered on Open Science Framework. ^13^

### Inclusion criteria

The Population, Concept and Context framework^14^ guided the development and scoping of this review to systematically address the research questions concerning HCD and its applications in designing DHDs used by older adults.

#### Population

The target population comprises all stakeholders involved in designing DHDs used in the care of older adults. The WHO age threshold for older adults (age 65+) was broadened to include participants aged 60 years and above, to extend the scope of insights into HCD for ageing populations.^2 3^

#### Concept

Studies were included if they reported the HCD of DHDs that incorporate self-reported data by the older adults. During screening, we broadened the term ‘dashboard’, where design context, visual elements and user interface descriptions were considered as indicative of a dashboard; or clinical decision-making tool features, even if the term ‘dashboard’ was not explicitly used. These included visualised summary displays, interactive data visualisation or longitudinal tracking interfaces. For this review, we treated HCD as an umbrella term (consistent with ISO 9241-210^7^) encompassing closely related approaches such as user-centred design (UCD) and participatory design that foreground end users throughout iterative design. Therefore, studies describing their approach as UCD were included where the reported methods aligned with these principles. We retained and extracted the original terminology used by study authors (UCD, participatory design, codesign or HCD), but grouped them at the analysis stage to map comparable participatory and iterative design activities.

#### Context

Interventions targeting the care of independent living and community-dwelling older adults who are able to self-report their data, whose needs differ from those in supported or institutional care settings. Partially assisted settings were considered if participants were able to engage independently in the HCD process. Ambulatory or clinician-facing dashboards developed for use in primary care settings are included if they support autonomy and ageing-in-place.^15^

### Search strategy

Search strategy was iteratively developed by the review team, based on the following keywords: ‘ageing’, ‘decision support systems’, ‘software design’, and ‘co-design’. CINAHL, MEDLINE, EMBASE, PsycINFO and Cochrane databases were searched from January 2012 through to 30 March 2026. Full search strategy is available in online supplemental appendix 2. Studies published in any language were searched, but only English articles were included.

### Study selection

**Figure 1:**
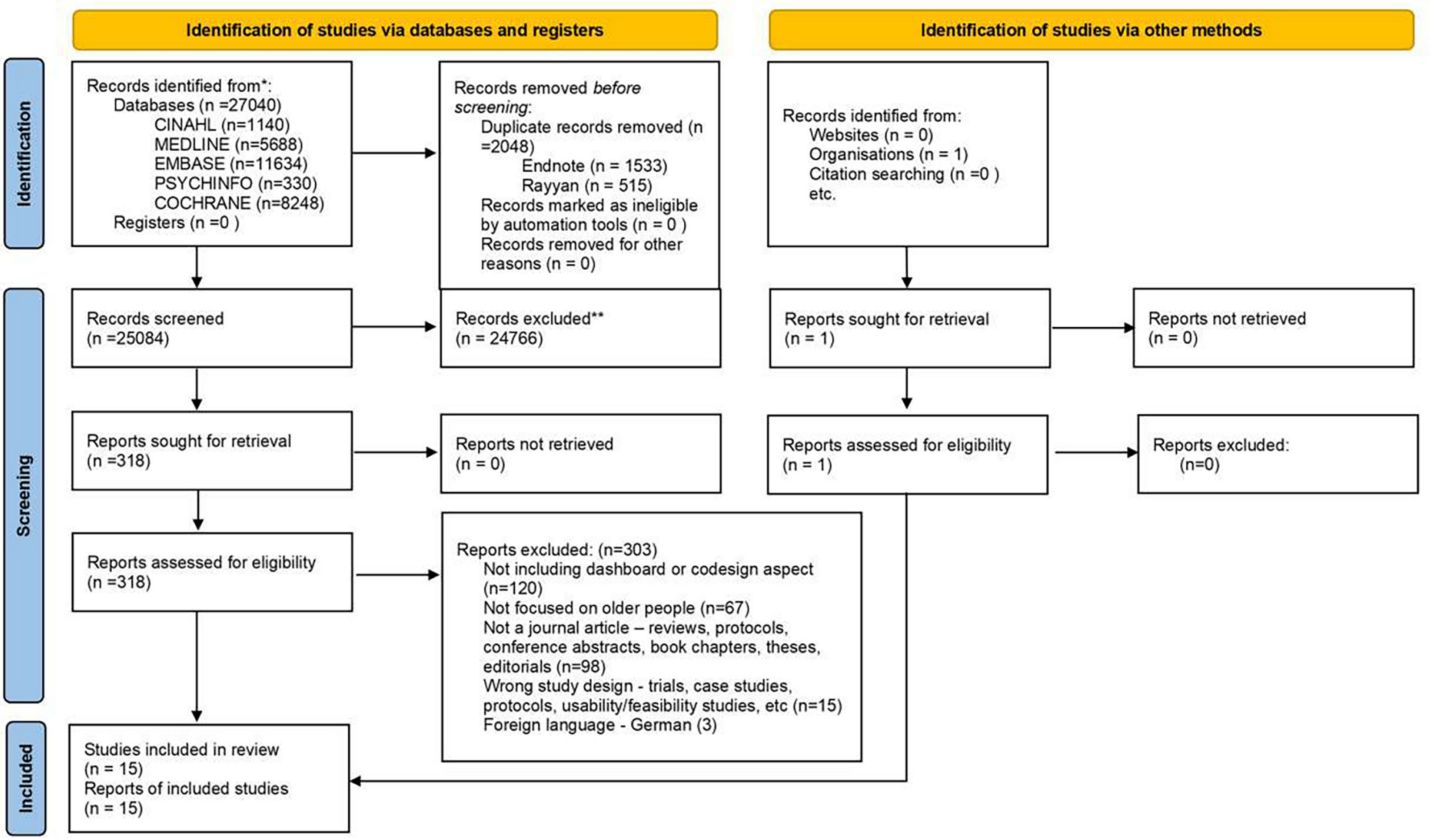
PRISMA 2020 flow diagram for new systematic reviews which included searches of databases, registers and other sources.

A total of 25 084 identified citations were uploaded into EndNote 20 (Clarivate Analytics, Pennsylvania, USA), and 1533 duplicates were identified and removed. A further 515 duplicates were removed when uploaded to Rayyan software by STAA, where abstract and title screening shortlisted a total of 318 articles. 20% of the abstracts and titles were also reviewed by additional reviewers BK and CF. A full-text review excluded 303 articles, with exclusion reasons recorded in Rayyan software, with 20% full texts also reviewed by the additional reviewers. Any disagreements were resolved through discussion within the review team. Exclusion reasons included the lack of HCD involvement, not focusing on self-management and the intervention not targeting the care of older adults. Experts were consulted, and an additional article was recommended, resulting in a total of 15 articles being included (figure 1).

### Critical appraisal of studies

The Mixed Methods Appraisal Tool^16^ was used to critically assess the quality of the mixed methods studies. Purely qualitative studies were assessed using the JBI checklist.^17^ In line with scoping review methods, this critical appraisal was conducted descriptively and did not inform the study inclusion or the interpretation of findings.^18^

### Data extraction

A modified version of the JBI data extraction tool, developed iteratively by the review team in Microsoft Excel, was used.^19^ Data included authors, year of publication, title, journal, study location, context (setting), participant details, research aims, questions, study design (qualitative, quantitative or mixed methods) and methodology. Results extracted included core design approach, HCD methods, tools, dashboard user, dashboard features and other theoretical frameworks. Where reported, data on factors related to access to technology, digital literacy and contextual or cultural influences on participation and use were extracted. See online supplemental appendix 3.

### Data analysis and synthesis of studies

Full texts were coded in NVivo 15 (STAA and BK), focusing on HCD frameworks, methods and tools, as well as the barriers and facilitators to HCD. A descriptive analysis is provided following a blended coding approach, guided by theme identification and development of a coding framework. Key findings were synthesised into narrative summaries, supported by visual representation.

**Figure 2:**
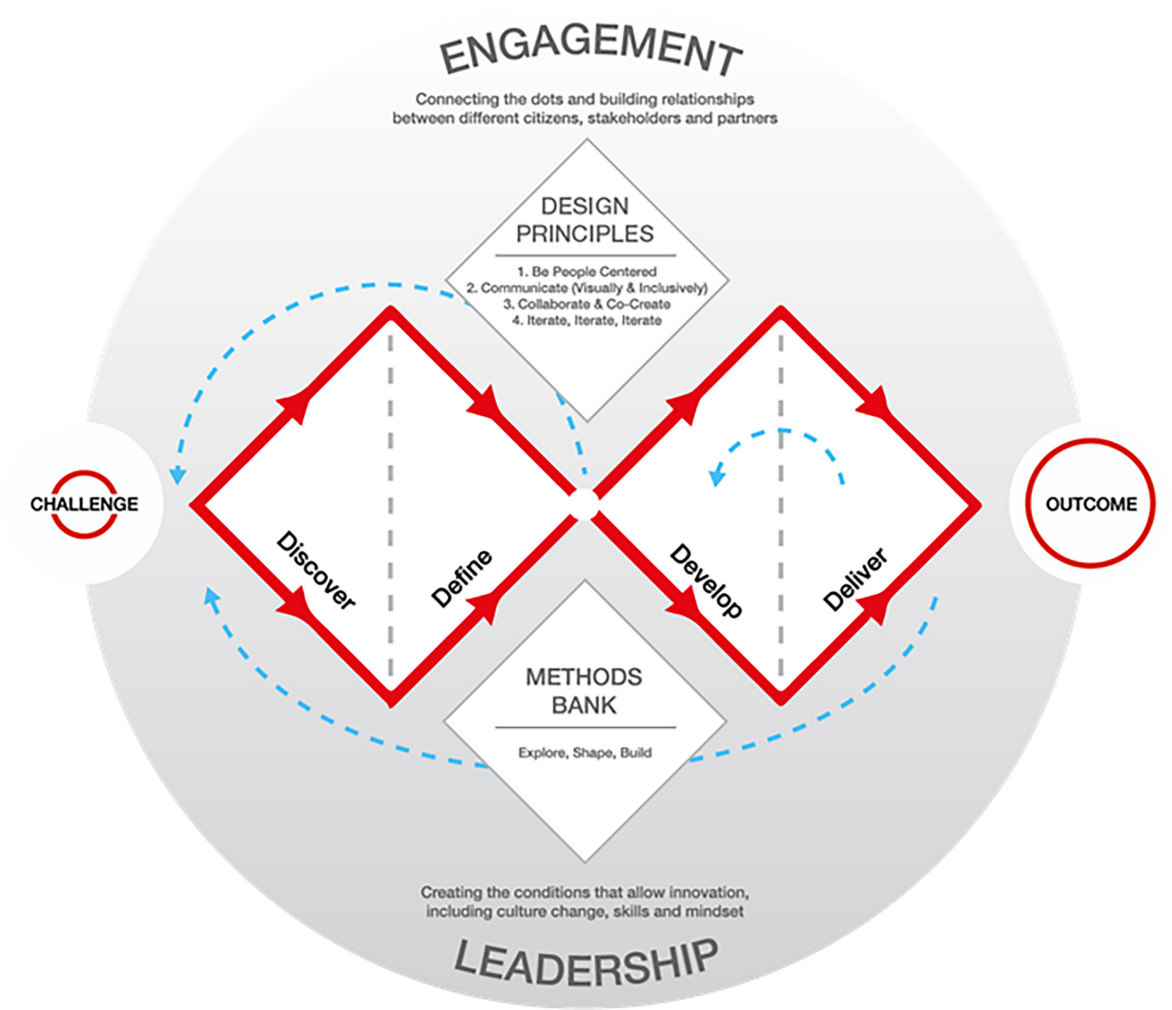
Double Diamond Framework (Design Council 2019).

A widely used framework for operationalising HCD, the Double Diamond framework, was used to map the HCD process (figure 2). It has four phases*: Discover, Define, Develop and Deliver,* which exercises iterative cycles of divergent and convergent thinking to inspire and develop a solution to a challenge.^20^,^22^ The first stage, ‘Discover’, addresses the problem by looking at the challenge as broadly as possible to understand the user’s perspective. The second phase, ‘Define’, focuses on all the data gathered in the previous phase and making sense of it. The third phase, ‘Develop’, involves iterative experimenting, creating and testing solutions. The final phase, ‘Deliver’ is about further refining the solution by testing it in a real-world setting.^23^

### Patient and public involvement

No patient and public involvement was undertaken while conducting this review.

## Results

### Study characteristics

The 15 included studies were published in 2019 or later, and only two were qualitative^24 25^ while the rest were mixed methods.^23 5 26^,^35^ All studies were of high methodological quality with common limitations relating to reflexivity in qualitative reporting and limited handling of divergence in mixed-methods integration (online supplemental appendix 4). First author affiliations by country included the USA (n=5, 33.3%),^326^,^28 31^ UK (n=2, 13.3%),^24 30^ Australia (n=1, 6.7%),^25^ Austria (n=1, 6.7%),^35^ Singapore (n=1, 6.7%),^34^ Canada (n=1, 6.7%),^33^ Spain (n=1, 6.7%),^32^ China (n=1, 6.7%),^29^ Belgium (n=1, 6.7%)^5^ and Ireland (n=1, 6.7%).^2^

Thirteen studies^23 5 24^,^30 33^ involved older adults from community settings, while two recruited older participants from academic tertiary care clinics.^31 32^ All studies except one involved healthcare professionals,^33^ and only one study did not involve caregivers.^5^ Two studies^24 31^ also involved policymakers. Older adults’ sample size ranged from 3 to 157 participants, and most of the studies had a higher proportion of older people.

Multiple stakeholders were involved through various HCD methods across the studies. These included older adults as end users, along with informal caregivers, healthcare professionals and other formal caregivers such as social workers, care home staff, rehabilitation assistants and occupational therapists. In addition, field experts and consultants including digital health researchers, behavioural experts, geriatric psychiatrists and decision scientists also contributed to the design and evaluation process. (Online supplemental material table 1) offers a comprehensive overview of the stakeholders reported in each study.

### Intervention and dashboard details

The included studies present interventions implemented across a diverse range of technological platforms including smartphone applications, tablet-based tools, web-based portals and integrated clinical dashboard interfaces. Mobile-based interventions were used to capture real-time and self-reported data and support use and sustained engagement, often incorporating wearables/sensors and cloud-based data storage.^524 27 29 30 33^,^35^ Web-based platforms and portals were commonly used to provide broader accessibility and support medication adherence and shared decision making by enabling remote access for healthcare professionals.^2 3 25 31^ Tablet apps were developed to accommodate accessibility needs including larger displays and simplified interfaces.^26 27^ Several of these interventions incorporated hybrid systems combining the mobile applications with web-based dashboards or clinical management, particularly in studies supporting both older adults and healthcare professionals,^24 27 30 32 34 35^ while two interventions had a separate smartphone application for the healthcare professionals.^29 30^

These platforms were designed to enable older adults to enhance overall quality of life through self-management of chronic conditions and medication adherence,^23 25 31^,^33^ promote physical activity and fall prevention^5 27 30 34^ and facilitate shared decision making for complex care needs.^24 28 31 35^ Some studies also included a specialised intervention for identifying mistreatment of older adults^26^ or applied holistic assessment and care planning frameworks to support comprehensive decision making.^24^ These digital health tools either have integrated dashboards explicitly^2 3 28 32^ or incorporated dashboard features to visualise health data, support behaviour change, enable remote monitoring by formal or informal caregivers and facilitate communication between older adults and their care networks.

These dashboards often provided a centralised overview of multiple patients or residents, displaying aggregated assessment results, tracking health metrics and symptoms over time and generating alerts for urgent needs or medication adherence.^5 30 33 35^ Clinician-focused interfaces facilitated clinical assessment, intervention recommendation and support communication regarding care plans and patient progress.^31 35^ Patient-facing dashboards offer personalised views of health data, allowing symptom monitoring, tracking physical activity and other health goals and visualising progress through intuitive charts and graphical summaries.^2732^,^34^ Other features included providing educational content, assisting with goal setting, displaying a Cardiac Health Score based on adherence, emoji-based emotional tagging, dual-format summaries to accommodate graph literacy, incorporating peer ranking for motivation, and generating printable summaries of personal information and decisions for users’ reference or to share with providers.^25 26 29 31 33^ (Online supplemental material table 2) provides details on the dashboard features reported across studies.

### Conceptual frameworks

The main approach used across all studies is UCD or HCD, emphasising iterative development and continuous stakeholder involvement, including older adults, formal and informal caregivers and healthcare or domain experts. However, while user-centred and participatory design principles were used across all studies, the explicit use and reporting of named co-design frameworks varied across the studies.

Only a few studies explicitly mentioned the use of a recognised framework. One study adapted the Sanders and Steppers co-design framework to guide their early-phase stakeholder-led development.^25^ Similarly, two studies explicitly reported using the structured design thinking methodology to guide the HCD process.^5 33^ Few other studies mentioned the use of UCD without detailing the specific framework used.^2 25 26 29 31 32 35^

Behaviour change frameworks were used to inform intervention content and functionality in several studies. These included the Intervention Mapping framework, Contemplation–Action–Maintenance (CAM) model,^29^ the Behaviour Change Wheel^5^ and Behaviour Change Taxonomy.^26 30^ One study implemented the Fogg Behaviour model operationalising motivation, ability and prompts through structured goal setting, feedback and gamified activity tracking to support active ageing.^34^ These strategies aimed to improve patient adherence to specific health management behaviours^2 3 5 29 30^ and facilitate the self-disclosure of critical health concerns.^26^

The Ottawa Decision Support Framework and Dartmouth’s Coproduction Design and Implementation Flow Model were employed to support patients’ informed decision-making related to choices in long-term care planning and collaborative management of advanced chronic illnesses.^28 31^ Several studies adopted the Medical Research Council (MRC) guidance for developing complex interventions.^5 24 30 32^

The agile method was used for technology development,^3 29 32^ and Grey’s self-management theory was employed to incorporate human-computer interaction principles to optimise usability for older adults.^33^ Furthermore, some design processes were guided by a logic model,^24^ principles of the nursing process^29^ or adherence to Web Content Accessibility Guidelines and British Broadcasting Corporation mobile accessibility guidelines to ensure inclusivity.^2^

### Methods for HCD

The four phases of the double diamond framework were used to map the HCD methods used in each study to examine the depth of the HCD practices as shown in (online supplemental material table 3).

*‘Discover’* *phase* had methods that were consistent across the studies, including literature review,^324 26 27 29^,^35^ interviews,^2 3 5 32^ focus groups^2 26 32^ and brainstorming and co-design workshops involving hands-on co-design activities with the participants.^2 24 25 28 33^

The *‘Define’ phase* was generally reported in less detail, with most studies briefly describing synthesis activities such as prioritising needs or translating insights into preliminary requirements.^326 28^,^32 35^ Two studies developed initial mock-ups based on the results from the discover phase.^30 35^ One study explicitly discussed synthesising evidence using a matrix developed through framework analysis^24^ while another study developed a conceptual model; mapped user needs to app features and prioritised their tasks.^33^ Three studies did not report the Define phase at all.^2 27 34^

The *‘Develop’ phase* involved iterative creation or refinement of mock-ups or prototypes^528^,^31 33^ and refinement of user scenarios and prototypes through interviews^3 27 30 33^ and workshops.^23 5 24^,^32 34^ However, all studies except one^28^ only described the co-design process at the level of the overall intervention, with limited specific attention to dashboard features, and lacked comprehensive reporting on the number of design iterations, development timelines and the evolution of dashboard prototypes.

Finally, the *‘Deliver’ phase* primarily emphasised prototype and usability testing over periods ranging from 3 weeks^30^ to eighteen months.^24^ Evaluation methods included think aloud sessions^5 26 31 34^ and surveys or structured questionnaires.^35 26 27 29 33^,^35^ Several studies also reported the real-world implementation of the prototypes in community or clinical settings.^24 28 32 34 35^ However, only a few incorporated analytics logs and usage metrics from the dashboards to track engagement or intervention outcomes.^2 3 27 29 34^ One focused exclusively on early-stage co-design and development and did not report Deliver stage implementation or evaluation activities.^25^

### Tools for HCD

Various technical design and support tools were used across the studies throughout the HCD process. To support human participation, studies employed mechanisms including technology training and ongoing user support,^2 26 27 30 31 34^ paper manuals,^2 24 27 31^ instruction videos,^24 31^ helpdesks^2 24^ and clinical support services^2 24 29^ to ensure smooth processes.

Online video conferencing and digital collaboration tools were used to facilitate remote workshops, meetings and evaluation activities. Zoom was the most commonly reported platform^24 25 33^ for this purpose while Microsoft Teams^35^ was also used. To support online engagement, Google Jamboard helped capture ideas,^24^ while Mural, Mentimeter and Padlet facilitated brainstorming, ranking and prioritisation activities.^35^

For development and design, mind mapping and wireframing were done through tools like XMind version 8,^29^ Axure,^29 33^ Sketch^33^ and Figma.^32 34^ Medium fidelity prototypes were designed on Flinto^29^ WordPress v2015^31^ and Figma^33^ while high fidelity, fully functional software was developed using PHP, Java and Nodejs.^27 29 35^ Supporting infrastructures included Linux, Docker and GitLab used as the operating environments,^29^ while system components were connected to the MYSQL^27^ or NoSQL-MongoDB^29^ databases.

Prototypes were demonstrated and tested across a range of hardware, including large display screens,^30^ Windows computers,^31^ smartphones^34 35^ and tablets.^2 5 26 27^ Interviews were captured using field notes,^3^ audio recordings^28 31 33^ and transcripts were thematically analysed using NVivo for Mac—V.11 and MAXQDA.^2^

### Facilitators and barriers to HCD

As the HCD process reported in the studies focused on the overall design of the digital interventions including DHDs, the facilitators and barriers from the broader HCD process are reported. A key facilitator was the use of multiple iterations of methods across the whole design process while engaging diverse stakeholders such as older adults, nurses, physicians, geriatricians, social workers, IT experts and advisory panels.^23 5 24^,^35^

Collaboration with community health programmes and care organisations was reported to facilitate the recruitment process and sustained engagement,^2 3 24 26 27 31 32 34 35^ as well as the use of design artefact tools such as design strategy maps, mock-ups and wireframes.^2931^,^33^

The adherence to evidence-based theoretical frameworks for behaviour change and decision making to guide content and technical development was also reported as a facilitator across studies.^526 29^,^31 34^ For technical development, agile development methodologies and modular or microservice architecture facilitated prompt modifications and responsiveness through clear communication between design and development teams.^3 29 32 35^ In more recent studies, remote participation via video conferencing and online collaboration platforms was also reported to improve accessibility of the HCD process.^2425 33^,^35^

Several barriers were reported from the start of the process during recruitment, with all studies except one^2^ struggling to achieve a diverse representation, which in turn limits the generalisability of the results.^35 24^,^35^ Maintaining engagement over time was also challenging, with several studies reporting user scepticism, limited motivation or a ‘not-for-me attitude’^3 27 34^ while others reported a lack of interest from the end users in using a digital intervention.^3 5 29^

Another barrier to the HCD process was the lack of digital literacy and data privacy concerns which affected both participant engagement and tool adoption.^325 30 33^,^35^ Fragmented healthcare systems were also mentioned as a barrier in identifying who would be responsible for identifying the unmet needs of the older people.^24^ Not involving all stakeholders in the HCD process from the start was also identified as a barrier, as it could impact the design and usability of the tool.^24 29^ In some cases, stakeholder input conflicted with evidence-based design, requiring the design team to navigate competing perspectives.^524 28^,^32^

Furthermore, resource or development constraints were also reported consistently across the studies in various phases of the HCD process, including budget limitations, short testing periods, selection bias and balancing agile development with scientific rigour.^23 5 24 26^,^32 34^ User limitations among older adults, such as lack of access to smartphones/larger screen devices, poor eyesight, limited finger dexterity and general lack of interest in computers also caused issues during the HCD process.^35 24 26^,^31 33^ Finally, short testing periods and a general lack of objective usage metrics made it difficult to systematically track the user interaction with the dashboards, limiting the understanding of real-time utility.^28 30 32 34^

#### Impact of the COVID-19 pandemic

Although this review did not take into consideration the Covid−19 pandemic at its conceptualisation, the inclusion of studies published from 2019 onwards requires the consideration of how COVID-19 may have influenced the HCD process. The included studies were published in 2019 or later and may reflect that the COVID-19 pandemic may have accelerated the development and adoption of DHDs in the care of older adults. Studies that commenced prior to the pandemic^2 27 31^ primarily focused more on in-person visits and face-to-face sessions. In contrast, research conducted during the pandemic focused on care delivery and management under lockdown conditions, emphasising the importance of real-time symptom monitoring and shared decision making when access to physical services was restricted.^28 29^ More recent studies highlight that the pandemic ‘accelerated the use of technology’ and demonstrated that older adults are now more ‘digital ready’ to engage with HCD of complex interventions.^24 25^

The most visible methodological impact was the transition of workshops, stakeholder meetings and interviews to online platforms, with studies relying on online recruitment strategies^3^ and videoconferencing tools for co-design and evaluation activities.^24 25 33 35^ Virtual collaboration tools^24 35^ and asynchronous feedback methods, such as written or email-based input,^24^ were incorporated to accommodate participants and maximise inclusivity. One study also integrated telehealth consultations conducted during the early COVID-19 period into its dashboard evaluation,^28^ while another adapted dashboard content to include COVID-19 symptom tracking, illustrating how pandemic conditions influenced both methodological approaches and intervention scope.^3^

## Discussion

### Findings

This review discussed the use of HCD in the development of DHDs for older adults. The studies reviewed were predominantly published after 2020, reflecting increased attention toward using technology to support health management in ageing populations. The use of interviews, focus groups, co-design workshops and repeated testing helped teams better understand users’ needs and iteratively refine DHDs to align them closely with user needs and preferences.

Across the studies, stakeholders included older adults, informal carers, healthcare professionals, technology and field experts, investigators, care home managers and leads and social workers. Their involvement was predominantly within the Discover Develop and Deliver phases, shaping needs identification, interface design and usability testing and refinement, while fewer studies demonstrated stakeholder involvement during the Define phase.

The limited reporting of the Define phase can significantly affect the outcomes of the HCD process. If user needs and insights are not strongly synthesised, it may reduce transparency in prioritisation and limit traceability between stakeholder inputs and final design decisions. In the context of this review, the reporting of the Define phase is particularly important to help reproduce, synthesise or evaluate the involvement of stakeholders while designing DHDs in the care of older adults.

DHDs are relatively new in managing the health of older adults^36^ and although researchers have started to address the acceptability and usability of such dashboards, there remains a gap in discussing the design and development of these dashboards.^37^ Previous literature has highlighted the limited application of HCD in dashboard development, with only a minority of studies explicitly referencing its use.^8^ While those studies focused primarily on public health dashboards in the USA, this distinction is necessary because clinician or policy-focused dashboards typically prioritise large-scale monitoring and decision making, whereas dashboards for older adults address accessibility, usability, emotional burden and self-management. Our findings extend this conversation by synthesising evidence specific to older adults and dashboards designed to support self-management and ageing-in-place.

Another recent study highlighting the need for HCD for developing dashboards in the care of older adults stresses the importance of involving older adults in the design of such DHDs to address their unique information needs.^38^ Our review adds to the literature by synthesising the existing evidence available on dashboards that have been developed using HCD involving older adults, as well as other stakeholders, experts from different fields, using mixed research methods.

Previous findings also underscore the intended audience of DHDs to be traditional stakeholder groups such as policy makers, healthcare professionals, researchers or the general public as the anticipated end users, while some studies do not share the intended audience.^8^ In comparison, our review reports studies designing DHDs specifically with the older population in mind to be able to present information according to older adults’ preferences and information-seeking behaviours.^38^ More recent studies published after 2024 demonstrate a shift beyond formative co-design toward quantitative usability testing and real-world evaluation, reflecting greater methodological maturity in the application of HCD to DHDs for older adults.

Another important influence across these studies was the impact of the COVID-19 pandemic on the design, recruitment and methodological execution.^3 32^ Although most studies did not explicitly discuss the impact of the pandemic, the findings suggest that it increased digital readiness among older adults and healthcare professionals, and overall in the design of health technologies for older adults.

**Limitations**:

The limitations identified in our review, such as inconsistencies in HCD frameworks, limited participant diversity and short testing durations, are consistent with challenges in broader dashboard literature noted previously.^8^ Social and economic health determinants, including access to technology, digital literacy and broader cultural or contextual influences, were only discussed narratively across studies and lacked consistent definitions. As a result, these factors could not be compared systematically across the interventions, limiting insights on how these could affect the engagement and use of DHDs.

The limited use of dashboard analytics constrains iterative refinement, real-world learning and the development of learning health systems, as evaluation relies mainly on self-reported data rather than continuous, system-generated evidence. The use of app-usage data and dashboard analytics to monitor user engagement and retention may be able to provide further insights into usability, engagement and user retention of these DHDs.

Our work advances the field by synthesising these challenges specifically within the context of care of older adults.

This scoping review has several potential limitations. Despite efforts to ensure the rigour of literature search, it may be possible that some relevant studies might be overlooked. The search focused on academic literature to report evidence-based dashboard design; hence, DHDs being used in the care of older adults without published information are possibly overlooked. Some included studies include older people below the WHO-recommended age range of 65, which could bias the results. Finally, variations in the detail of the studies may have limited the robustness of the analysis for practical guidance on HCD-based DHD design in care for older adults.

#### Recommendations for future research

Future studies should aim for greater theoretical coherence by explicitly adopting and reporting validated HCD frameworks. Research funding and study design must account for structural barriers to stakeholder participation, including equitable compensation for healthcare professionals and support for underrepresented populations.^31^ Further inquiry is needed into how ageing-related stigma, digital literacy and household roles shape user engagement. The CAM model proposed by Chen et al. offers a promising lens to explore these co-constructed dynamics.^29^ The in-app usage analytics to complement traditional usability testing can inform more responsive, data-driven design iterations and identify friction points that may not surface in interviews or surveys, especially given memory-related limitations in older populations. This shift from static to dynamic evaluation can be critical in improving long-term adherence and tailoring interventions more precisely. Hedonic and sociability experiences should be systematically integrated into intervention design to sustain long-term engagement and mitigate emotional burden, particularly in end-of-life and chronic care contexts.^3 27^ Developing scalable and sustainable models of participatory engagement, including asynchronous feedback tools and hybrid co-design models, may enhance reach and continuity in HCD implementation.

## Conclusion

This scoping review explored how HCD has been used in the development of DHDs for older adults. Most studies were conducted after 2020, showing growing interest in technology-supported care for ageing populations. While the review included studies from various countries, most were from Europe and North America, raising concerns about the lack of representation or healthcare quality in low- and middle-income countries.

The review highlighted strengths in how researchers engaged older adults and other stakeholders across all stages of design. The use of interviews, focus groups, co-creation and iterative testing appears to help ensure that the technologies reflected the needs and preferences of users. This was associated with positive outcomes, including potential improvements in usability, self-management and emotional engagement, with the most recent studies also reporting structured usability evaluation and early real-world implementation. However, many studies did not use a consistent or validated framework to guide the HCD process, which limits how easily their findings can be applied in other settings.

There are also areas that need more attention. These include limited use of built-in usage analytics to understand how DHDs are used over time, and challenges in involving healthcare professionals and harder-to-reach groups. Cultural and emotional factors, such as fear, stigma and low digital confidence also affected how older adults engaged with these technologies.

Overall, this review supports the value of HCD in designing more meaningful and effective health tools for older people. To improve future work, studies should use clearer frameworks, consider cultural and emotional contexts, and explore how digital tools are used in real-world settings over time. Doing so can support the development of more inclusive, engaging and sustainable health technologies.

## Supplementary material

10.1136/bmjopen-2025-113525 online supplemental appendix 1

10.1136/bmjopen-2025-113525 online supplemental appendix 2

10.1136/bmjopen-2025-113525 online supplemental appendix 3

10.1136/bmjopen-2025-113525 online supplemental appendix 4

10.1136/bmjopen-2025-113525 online supplemental table 1

10.1136/bmjopen-2025-113525 online supplemental table 2

10.1136/bmjopen-2025-113525 online supplemental table 3

## Data Availability

All data relevant to the study are included in the article or uploaded as supplementary information.
