# Supplementary material for: Human-centred design of digital health dashboards in care of older adults: a scoping review
Source: BMJ Open. 2026 Jul 17;16(7):e113525. doi: 10.1136/bmjopen-2025-113525 (PMC13384139; doi:10.1136/bmjopen-2025-113525)
Supplement: online supplemental appendix 2 [file bmjopen-16-7-s002.docx]

## Appendix 2: Search strategy

### PsychInfo

| 1 | Health Services for the Aged/ |
| --- | --- |
| 2 | (geriatric* or elder* or old age or old* adult* or senior* or old* patient* or old* person* or old* people* or retire* or aging or ageing or age-related).m_titl. |
| 3 | (geriatric* or elder* or old age or old* adult* or senior* or old* patient* or old* person* or old* people* or retire* or aging or ageing or age-related).mp. [mp=title, abstract, heading word, table of contents, key concepts, original title, tests & measures, mesh word] |
| 4 | Aged.mp. or "Aged, 80 and over"/ or Aged/ or Health Services for the Aged/ |
| 5 | Aged, 80 and over/ or Adult/ or Aging/ or ageing.mp. or Aged/ |
| 6 | Decision Making/ or Information Systems/ or Decision Support Systems, Clinical/ or Software/ or Diagnosis, Computer-Assisted/ |
| 7 | Telemedicine/ or Artificial Intelligence/ or Decision Support Systems, Management/ |
| 8 | Decision Support Techniques/ or Decision Making/ |
| 9 | Software Design.mp. or Software Design/ |
| 10 | (dashboard or interface design or interaction design or software design or decision support or decision-support or decision making or scorecard* or analytics or computeri?ed reminder* or usability or usability principles or HCI or human-computer interaction or CHI or computer-human interaction or informatoin design or cognitive engineering or adaptive display or congitive workload or cognitive efford or UI or user interface or human interface or user-centered or user-centred or human-centered or human-centred or cognitive analysis or cognitive task analysis).m_titl. |
| 11 | (dashboard or interface design or interaction design or software design or decision support or decision-support or decision making or scorecard* or analytics or computeri?ed reminder* or usability or usability principles or HCI or human-computer interaction or CHI or computer-human interaction or informatoin design or cognitive engineering or adaptive display or congitive workload or cognitive efford or UI or user interface or human interface or user-centered or user-centred or human-centered or human-centred or cognitive analysis or cognitive task analysis).mp. [mp=title, abstract, heading word, table of contents, key concepts, original title, tests & measures, mesh word] |
| 12 | (co-design or participatory design or collaborative design).mp. [mp=title, abstract, heading word, table of contents, key concepts, original title, tests & measures, mesh word] |
| 13 | (user-centered design or human-centered design or ergonomic design).mp. [mp=title, abstract, heading word, table of contents, key concepts, original title, tests & measures, mesh word] |
| 14 | (stakeholder engag* or patient partner* or patient cent* care or client cent* care or person cent* care).mp. [mp=title, abstract, heading word, table of contents, key concepts, original title, tests & measures, mesh word] |
| 15 | (shared decision making or patient involvement or involvement in decisions or sdm).mp. [mp=title, abstract, heading word, table of contents, key concepts, original title, tests & measures, mesh word] |
| 16 | 12 or 13 or 14 or 15 |
| 17 | 1 or 2 or 3 or 4 or 5 |
| 18 | 6 or 7 or 8 or 9 or 10 or 11 |
| 19 | 16 and 17 and 18 |
| 20 | limit 19 to ("remove medline records" and yr="2012 - 2024") |

### Embase

| 1 | Health Services for the Aged/ |
| --- | --- |
| 2 | (geriatric* or elder* or old age or old* adult* or senior* or old* patient* or old* person* or old* people* or retire* or aging or ageing or age-related).m_titl. |
| 3 | (geriatric* or elder* or old age or old* adult* or senior* or old* patient* or old* person* or old* people* or retire* or aging or ageing or age-related).mp. [mp=title, abstract, heading word, drug trade name, original title, device manufacturer, drug manufacturer, device trade name, keyword heading word, floating subheading word, candidate term word] |
| 4 | Aged.mp. or "Aged, 80 and over"/ or Aged/ or Health Services for the Aged/ |
| 5 | Aged, 80 and over/ or Adult/ or Aging/ or ageing.mp. or Aged/ |
| 6 | Decision Making/ or Information Systems/ or Decision Support Systems, Clinical/ or Software/ or Diagnosis, Computer-Assisted/ |
| 7 | Telemedicine/ or Artificial Intelligence/ or Decision Support Systems, Management/ |
| 8 | Decision Support Techniques/ or Decision Making/ |
| 9 | Software Design.mp. or Software Design/ |
| 10 | (dashboard or interface design or interaction design or software design or decision support or decision-support or decision making or scorecard* or analytics or computeri?ed reminder* or usability or usability principles or HCI or human-computer interaction or CHI or computer-human interaction or informatoin design or cognitive engineering or adaptive display or congitive workload or cognitive efford or UI or user interface or human interface or user-centered or user-centred or human-centered or human-centred or cognitive analysis or cognitive task analysis).m_titl. |
| 11 | (dashboard or interface design or interaction design or software design or decision support or decision-support or decision making or scorecard* or analytics or computeri?ed reminder* or usability or usability principles or HCI or human-computer interaction or CHI or computer-human interaction or informatoin design or cognitive engineering or adaptive display or congitive workload or cognitive efford or UI or user interface or human interface or user-centered or user-centred or human-centered or human-centred or cognitive analysis or cognitive task analysis).mp. [mp=title, abstract, heading word, drug trade name, original title, device manufacturer, drug manufacturer, device trade name, keyword heading word, floating subheading word, candidate term word] |
| 12 | (co-design or participatory design or collaborative design).mp. [mp=title, abstract, heading word, drug trade name, original title, device manufacturer, drug manufacturer, device trade name, keyword heading word, floating subheading word, candidate term word] |
| 13 | (user-centered design or human-centered design or ergonomic design).mp. [mp=title, abstract, heading word, drug trade name, original title, device manufacturer, drug manufacturer, device trade name, keyword heading word, floating subheading word, candidate term word] |
| 14 | (stakeholder engag* or patient partner* or patient cent* care or client cent* care or person cent* care).mp. [mp=title, abstract, heading word, drug trade name, original title, device manufacturer, drug manufacturer, device trade name, keyword heading word, floating subheading word, candidate term word] |
| 15 | (shared decision making or patient involvement or involvement in decisions or sdm).mp. [mp=title, abstract, heading word, drug trade name, original title, device manufacturer, drug manufacturer, device trade name, keyword heading word, floating subheading word, candidate term word] |
| 16 | 12 or 13 or 14 or 15 |
| 17 | 1 or 2 or 3 or 4 or 5 |
| 18 | 6 or 7 or 8 or 9 or 10 or 11 |
| 19 | 16 and 17 and 18 |
| 20 | limit 19 to ("remove medline records" and yr="2012 - 2024") |

### MEDLINE

| 1 | Health Services for the Aged/ |
| --- | --- |
| 2 | (geriatric* or elder* or old age or old* adult* or senior* or old* patient* or old* person* or old* people* or retire* or aging or ageing or age-related).m_titl. |
| 3 | (geriatric* or elder* or old age or old* adult* or senior* or old* patient* or old* person* or old* people* or retire* or aging or ageing or age-related).mp. [mp=title, book title, abstract, original title, name of substance word, subject heading word, floating sub-heading word, keyword heading word, organism supplementary concept word, protocol supplementary concept word, rare disease supplementary concept word, unique identifier, synonyms, population supplementary concept word, anatomy supplementary concept word] |
| 4 | Aged.mp. or "Aged, 80 and over"/ or Aged/ or Health Services for the Aged/ |
| 5 | Aged, 80 and over/ or Adult/ or Aging/ or ageing.mp. or Aged/ |
| 6 | Decision Making/ or Information Systems/ or Decision Support Systems, Clinical/ or Software/ or Diagnosis, Computer-Assisted/ |
| 7 | Telemedicine/ or Artificial Intelligence/ or Decision Support Systems, Management/ |
| 8 | Decision Support Techniques/ or Decision Making/ |
| 9 | Software Design.mp. or Software Design/ |
| 10 | (dashboard or interface design or interaction design or software design or decision support or decision-support or decision making or scorecard* or analytics or computeri?ed reminder* or usability or usability principles or HCI or human-computer interaction or CHI or computer-human interaction or informatoin design or cognitive engineering or adaptive display or congitive workload or cognitive efford or UI or user interface or human interface or user-centered or user-centred or human-centered or human-centred or cognitive analysis or cognitive task analysis).m_titl. |
| 11 | (dashboard or interface design or interaction design or software design or decision support or decision-support or decision making or scorecard* or analytics or computeri?ed reminder* or usability or usability principles or HCI or human-computer interaction or CHI or computer-human interaction or informatoin design or cognitive engineering or adaptive display or congitive workload or cognitive efford or UI or user interface or human interface or user-centered or user-centred or human-centered or human-centred or cognitive analysis or cognitive task analysis).mp. [mp=title, book title, abstract, original title, name of substance word, subject heading word, floating sub-heading word, keyword heading word, organism supplementary concept word, protocol supplementary concept word, rare disease supplementary concept word, unique identifier, synonyms, population supplementary concept word, anatomy supplementary concept word] |
| 12 | (co-design or participatory design or collaborative design).mp. [mp=title, book title, abstract, original title, name of substance word, subject heading word, floating sub-heading word, keyword heading word, organism supplementary concept word, protocol supplementary concept word, rare disease supplementary concept word, unique identifier, synonyms, population supplementary concept word, anatomy supplementary concept word] |
| 13 | (user-centered design or human-centered design or ergonomic design).mp. [mp=title, book title, abstract, original title, name of substance word, subject heading word, floating sub-heading word, keyword heading word, organism supplementary concept word, protocol supplementary concept word, rare disease supplementary concept word, unique identifier, synonyms, population supplementary concept word, anatomy supplementary concept word] |
| 14 | (stakeholder engag* or patient partner* or patient cent* care or client cent* care or person cent* care).mp. [mp=title, book title, abstract, original title, name of substance word, subject heading word, floating sub-heading word, keyword heading word, organism supplementary concept word, protocol supplementary concept word, rare disease supplementary concept word, unique identifier, synonyms, population supplementary concept word, anatomy supplementary concept word] |
| 15 | (shared decision making or patient involvement or involvement in decisions or sdm).mp. [mp=title, book title, abstract, original title, name of substance word, subject heading word, floating sub-heading word, keyword heading word, organism supplementary concept word, protocol supplementary concept word, rare disease supplementary concept word, unique identifier, synonyms, population supplementary concept word, anatomy supplementary concept word] |
| 16 | 12 or 13 or 14 or 15 |
| 17 | 1 or 2 or 3 or 4 or 5 |
| 18 | 6 or 7 or 8 or 9 or 10 or 11 |
| 19 | 16 and 17 and 18 |
| 20 | limit 19 to ("remove medline records" and yr="2012 - 2024") |

### CINAHL Plus

| # | Query | Limiters/Expanders |
| --- | --- | --- |
| S31 | S18 AND S24 AND S29 | Limiters - Publication Date: 20120101-20241231 |
|  |  | Expanders - Apply equivalent subjects |
|  |  | Search modes - Proximity |
| S30 | S18 AND S24 AND S29 | Expanders - Apply equivalent subjects |
|  |  | Search modes - Proximity |
|  |  |  |
| S29 | S25 OR S26 OR S27 OR S28 | Expanders - Apply equivalent subjects |
|  |  | Search modes - Proximity |
|  |  |  |
| S28 | TI ( shared decision making or patient involvement or involvement in decisions or sdm ) OR AB ( shared decision making or patient involvement or involvement in decisions or sdm ) OR MW ( shared decision making or patient involvement or involvement in decisions or sdm ) OR MJ ( shared decision making or patient involvement or involvement in decisions or sdm ) | Limiters - Publication Date: 20220101-20241231 |
|  |  | Expanders - Apply equivalent subjects |
|  |  | Search modes - Proximity |
| S27 | TI ( stakeholder engag* or patient partner* or patient cent* care or client cent* care or person cent* care ) OR AB ( stakeholder engag* or patient partner* or patient cent* care or client cent* care or person cent* care ) OR MJ ( stakeholder engag* or patient partner* or patient cent* care or client cent* care or person cent* care ) OR MW ( stakeholder engag* or patient partner* or patient cent* care or client cent* care or person cent* care ) | Limiters - Publication Date: 20220101-20241231 |
|  |  | Expanders - Apply equivalent subjects |
|  |  | Search modes - Proximity |
| S26 | TI ( user-centered design or human-centered design or ergonomic design ) OR AB ( user-centered design or human-centered design or ergonomic design ) | Expanders - Apply equivalent subjects |
|  |  | Search modes - Proximity |
|  |  |  |
| S25 | TI ( co-design or participatory design or collaborative design ) OR AB ( co-design or participatory design or collaborative design ) | Expanders - Apply equivalent subjects |
|  |  | Search modes - Proximity |
|  |  |  |
| S24 | S19 OR S20 OR S21 OR S22 OR S23 | Expanders - Apply equivalent subjects |
|  |  | Search modes - Proximity |
|  |  |  |
| S23 | TI ( dashboard or interface design or interaction design or software design or decision support or decision-support or decision making or scorecard* or analytics or computeri?ed reminder* or usability or usability principles or HCI or human-computer interaction or CHI or computer-human interaction or informatoin design or cognitive engineering or adaptive display or congitive workload or cognitive efford or UI or user interface or human interface or user-centered or user-centred or human-centered or human-centred or cognitive analysis or cognitive task analysis ) OR AB ( dashboard or interface design or interaction design or software design or decision support or decision-support or decision making or scorecard* or analytics or computeri?ed reminder* or usability or usability principles or HCI or human-computer interaction or CHI or computer-human interaction or informatoin design or cognitive engineering or adaptive display or congitive workload or cognitive efford or UI or user interface or human interface or user-centered or user-centred or human-centered or human-centred or cognitive analysis or cognitive task analysis ) | Expanders - Apply equivalent subjects |
|  |  | Search modes - Proximity |
|  |  |  |
| S22 | MH Software Design | Expanders - Apply equivalent subjects |
|  |  | Search modes - Proximity |
|  |  |  |
| S21 | MH Decision Support Techniques | Expanders - Apply equivalent subjects |
|  |  | Search modes - Proximity |
|  |  |  |
| S20 | MM Decision Support Systems, Management | Expanders - Apply equivalent subjects |
|  |  | Search modes - Proximity |
|  |  |  |
| S19 | MM Decision Support Systems, Clinical | Expanders - Apply equivalent subjects |
|  |  | Search modes - Proximity |
|  |  |  |
| S18 | S14 OR S15 OR S16 OR S17 | Expanders - Apply equivalent subjects |
|  |  | Search modes - Proximity |
|  |  |  |
| S17 | MH Aging | Expanders - Apply equivalent subjects |
|  |  | Search modes - Proximity |
|  |  |  |
| S16 | MH Aged+ | Expanders - Apply equivalent subjects |
|  |  | Search modes - Proximity |
|  |  |  |
| S15 | TI ( geriatric? or elder* or old age or old* adult? or senior? or old* patient? or old* person? or old* people? or retire* or aging or ageing or age-related ) OR AB ( geriatric? or elder* or old age or old* adult? or senior? or old* patient? or old* person? or old* people? or retire* or aging or ageing or age-related ) | Expanders - Apply equivalent subjects |
|  |  | Search modes - Proximity |
|  |  |  |
| S14 | MH Health Services for Older Persons | Expanders - Apply equivalent subjects |
|  |  | Search modes - Proximity |
|  |  |  |
| S13 | S12 | Limiters - Publication Date: 20220101-20241231 |
|  |  | Expanders - Apply equivalent subjects |
|  |  | Search modes - Proximity |
| S12 | S5 AND S11 | Expanders - Apply equivalent subjects |
|  |  | Search modes - Proximity |
|  |  |  |
| S11 | S6 OR S7 OR S8 OR S9 OR S10 | Expanders - Apply equivalent subjects |
|  |  | Search modes - Proximity |
|  |  |  |
| S10 | TI ( dashboard or interface design or interaction design or software design or decision support or decision-support or decision making or scorecard* or analytics or computeri?ed reminder* or usability or usability principles or HCI or human-computer interaction or CHI or computer-human interaction or informatoin design or cognitive engineering or adaptive display or congitive workload or cognitive efford or UI or user interface or human interface or user-centered or user-centred or human-centered or human-centred or cognitive analysis or cognitive task analysis ) OR AB ( dashboard or interface design or interaction design or software design or decision support or decision-support or decision making or scorecard* or analytics or computeri?ed reminder* or usability or usability principles or HCI or human-computer interaction or CHI or computer-human interaction or informatoin design or cognitive engineering or adaptive display or congitive workload or cognitive efford or UI or user interface or human interface or user-centered or user-centred or human-centered or human-centred or cognitive analysis or cognitive task analysis ) | Expanders - Apply equivalent subjects |
|  |  | Search modes - Proximity |
|  |  |  |
| S9 | MH Software Design | Expanders - Apply equivalent subjects |
|  |  | Search modes - Proximity |
|  |  |  |
| S8 | MH Decision Support Techniques | Expanders - Apply equivalent subjects |
|  |  | Search modes - Proximity |
|  |  |  |
| S7 | MM Decision Support Systems, Management | Expanders - Apply equivalent subjects |
|  |  | Search modes - Proximity |
|  |  |  |
| S6 | MM Decision Support Systems, Clinical | Expanders - Apply equivalent subjects |
|  |  | Search modes - Proximity |
|  |  |  |
| S5 | S1 OR S2 OR S3 OR S4 | Expanders - Apply equivalent subjects |
|  |  | Search modes - Proximity |
|  |  |  |
| S4 | MH Aging | Expanders - Apply equivalent subjects |
|  |  | Search modes - Proximity |
|  |  |  |
| S3 | MH Aged+ | Expanders - Apply equivalent subjects |
|  |  | Search modes - Proximity |
|  |  |  |
| S2 | TI ( geriatric? or elder* or old age or old* adult? or senior? or old* patient? or old* person? or old* people? or retire* or aging or ageing or age-related ) OR AB ( geriatric? or elder* or old age or old* adult? or senior? or old* patient? or old* person? or old* people? or retire* or aging or ageing or age-related ) | Expanders - Apply equivalent subjects |
|  |  | Search modes - Proximity |
|  |  |  |
| S1 | MH Health Services for Older Persons | Expanders - Apply equivalent subjects |
|  |  | Search modes - Proximity |
|  |  |  |

### Cochrane Library

| ID | Search |
| --- | --- |
| #1 | (Health Services for the Aged):ti,ab,kw (Word variations have been searched) |
| #2 | (geriatric* or elder* or old age or old* adult* or senior* or old* patient* or old* person* or old* people* or retire* or aging or ageing or age-related):ti,ab,kw |
| #3 | (Aged or Aged 80 and over or Adult or Aging or ageing):ti,ab,kw |
| #4 | {OR #1 - #3} |
| #5 | (Decision Making or Information Systems or Decision Support Systems, Clinical or Software or Diagnosis, Computer-Assisted):ti,ab,kw |
| #6 | (Telemedicine or Artificial Intelligence or Decision Support Systems, Management):ti,ab,kw |
| #7 | (Decision Support Techniques or Decision Making):ti,ab,kw |
| #8 | (Software Design.mp. or Software Design):ti,ab,kw |
| #9 | (dashboard or interface design or interaction design or software design or decision support or decision-support or decision making or scorecard* or analytics or computeri?ed reminder* or usability or usability principles or HCI or human-computer interaction or CHI or computer-human interaction or informatoin design or cognitive engineering or adaptive display or congitive workload or cognitive efford or UI or user interface or human interface or user-centered or user-centred or human-centered or human-centred or cognitive analysis or cognitive task analysis):ti,ab,kw |
| #10 | {OR #5 - #9} |
| #11 | (co-design or participatory design or collaborative design):ti,ab,kw |
| #12 | (user cent* design or human cent* design or ergonomic design):ti,ab,kw |
| #13 | stakeholder engag* or patient partner* or patient cent* care or client cent* care or person cent* care |
| #14 | (shared decision making or patient involvement or involvement in decisions or sdm):ti,ab,kw |
| #15 | {OR #11 - #14} |
| #16 | #4 AND #10 AND #15 with Cochrane Library publication date Between Jan 2012 and Dec 2024, in Cochrane Reviews, Cochrane Protocols (Word variations have been searched) |
