# Supplementary material for: Human-centred design of digital health dashboards in care of older adults: a scoping review
Source: BMJ Open. 2026 Jul 17;16(7):e113525. doi: 10.1136/bmjopen-2025-113525 (PMC13384139; doi:10.1136/bmjopen-2025-113525)
Supplement: online supplemental appendix 3 [file bmjopen-16-7-s003.docx]

## Appendix 3: Data extraction instrument

### Scoping Review Details

| **Scoping Review Details** | | |
| --- | --- | --- |
| Scoping Review title: | Human-centred design of Digital Health Dashboards in care of Older Adults: A Scoping Review | |
| Review objective/s: | To map the literature available on HCD methodologies for developing DHDs that incorporate self-reported data on older adults, co-design challenges and potential strategies. | |
| Review question/s: | 1. What conceptual frameworks are used when conducting HCD for designing DHDs for self-management of health in care older adult data? 2. What are the HCD methods when designing these dashboards? 3. What are the challenges and facilitators to HCD? | |
| **Inclusion/Exclusion Criteria** | **Inclusion** | **Exclusion** |
| Population | All stakeholders, including older adults (mean age 60 or above), family members, experts, researchers, healthcare professionals and other formal and informal caregivers involved in the design of Digital Health Dashboards (DHDs) benefiting community-dwelling older adults. The WHO age threshold for older adults (age 65+) was broadened to include participants aged 60 years and above, to extend the scope of insights into HCD for ageing populations | Interventions that are not focused on benefitting older adults through self-reporting of health |
| Concept | The central concept of this review is Human Centred Design (HCD) of interventions that involved self reporting of symptoms by older people and featured, enabled, or implied dashboard functionalities—even if the term “dashboard” was not explicitly used. This applied to tools providing visualisation, decision support, or summary overviews relevant to the care of older adults. During screening design context, visual elements, and user interface descriptions were considered as indicative of dashboard development where appropriate. | Studies were excluded if they did not mention any aspect of HCD, did not mention symptoms being self reported by older people, and did not incorporate any dashboard related features. |
| Context | The included studies explore the development of HCD-based DHDS in diverse settings, with a focus on home, community and independent living environments. Studies involving partially assisted settings were considered if participants were able to engage independently in the HCD process. Clinician-facing dashboards developed for use in primary care settings are included if they support autonomy and ageing-in-place or if the design process accounts for broader patient-centred goals aligned with independent living. | Clinician focused dashboards were excluded unless the symptoms were reported by older adults prior to their appointment |
| Types of evidence source | Any published articles/ theses/grey literature, reporting all types of study designs employing qualitative, quantitative, and mixed methods approaches | Literature/systematic reviews - reference lists screened to identify relevant studies.  Languages other than English |

### Source Details

| **Author, year** | **Title** | **Journal** | **Location** | **Context (setting)** | **Participants** | **Research Aims** | **Research Questions** | **Study Design** | **Study Duration** | **Methodology** | |
| --- | --- | --- | --- | --- | --- | --- | --- | --- | --- | --- | --- |
| Abujarad, 2021 | Development and usability evaluation of VOICES: A digital health tool to identify elder mistreatment. | Journal of the American Geriatrics Society -Clinical Investigations | New Haven, Connecticut, USA. | Participant recruitment took place in community settings via local community centers and aging service providers (Agency on Aging of South Central Connecticut). | Age Range: Primarily 65-84 years old. Gender Distribution: Majority female (83.3%). Ethnicity: Mostly White/Caucasian (91.7%). Comfort with Technology: Varying levels of experience with computers. Financial Means: Most had social security (83.3%) and other financial means. Caregiving Status: Six participants were caregivers. Care Needs: Considering long-term care services within the next year. | To describe the development and usability evaluation of VOICES, a self-administrated digital health tool that screens, educates, and motivates older adults to self-report elder mistreatment. | RQ1: How do older adults perceive and react to the concept of using a tablet-based tool to screen for elder mistreatment in the emergency department setting? RQ2: How easy, acceptable, and engaging is the VOICES tool for older adults to use? RQ3: How well do older adults understand the information and content provided by the VOICES tool? RQ4: How does the VOICES tool affect the emotional state and self-efficacy of older adults regarding elder mistreatment? | Mixed Methods, primarily quantitative, using numerical data and statistical analysis to measure the usability and acceptability of the VOICES tool. Qualitative focus groups and open-ended feedback to gather user input and suggestions for improvement. | Not specified | User-Centred Design (UCD): Involving end users and stakeholders in all stages of the design process, including requirement gathering, conceptual model design, focus groups and interviews, prototyping and mock-ups, tool development, and usability evaluation. | |
| Afolabi, 2025 | First steps in co‑designing an online patient decision support tool for enhanced medication management in older people | Australasian Journal on Ageing | Sydney, New South Wales, Australia (multi-state involvement) | Stakeholder committee consultations via videoconferencing; older adults and carers recruited through a national frailty network; multi-disciplinary input from 3 Australian states. | *Stakeholder Committee (n=9):* 2 older adults (65+ years), 1 informal carer, 1 pharmacist, 1 nurse, 1 geriatrician, 1 general practitioner, 2 digital health researchers (across NSW, SA, QLD) | To develop a preliminary online medication management patient decision support (PDS) tool for older people using a co‑design approach with older adults, caregivers, and healthcare professionals. | RQ1. How can a stakeholder‑led co‑design process inform the development of an online patient decision support tool to support medication management for older people?  RQ2. What features, content, and design considerations do older adults, carers, and clinicians prioritise in an online medication management decision support tool? | Qualitative co‑design study (stakeholder engagement, iterative prototype development); descriptive reporting of early co‑design phases (no clinical trial). | Not specified. | Co‑design methodology (adapted Sanders & Stappers framework): multi-stakeholder involvement (older people, carer, clinicians, researchers) through structured meetings and feedback; iterative MVP development guided by user input; brief qualitative content analysis of meeting notes and correspondence to inform design. | |
| Bao, 2025 | Design and Evaluation of a Digital Health App (SingaporeWALK) for Active Aging: Pre‑Post Intervention Study | JMIR Formative Research | Singapore | Participant recruitment and intervention conducted in senior activity centres/community centres facilitating group‑based physical activity and social interaction. | Older adults: 48 participants completed the intervention (51 recruited).  Age range: 60–85 years.  Community‑dwelling older adults proficient in English or Chinese | To design and evaluate a culturally inclusive digital health app to promote active aging among older adults and reduce barriers to technology adoption through participatory design and community‑based implementation. | RQ1. How can a participatory design approach be used to develop a culturally inclusive digital health app to promote active ageing among older adults?  RQ2. Does use of the SGWALK app improve usability and technology acceptance among older adults?  RQ3. Does participation in the SGWALK intervention lead to changes in physical activity, nutrition‑related behaviours, and mental well‑being?  RQ4. How do different implementation supports (exercise only, exergames, coach support, peer support) influence engagement and outcomes? | Mixed‑methods with qualitative participatory design workshops; and quantitative usability testing (SUS) | 4‑week intervention period. | Participatory design methodology involving iterative testing cycles with older adults; formative usability evaluation; integration of user feedback into interface, navigation, and data visualisation design; pre‑post quantitative assessment of physical activity, mental well‑being, nutrition behaviour, and technology acceptance. | |
| Cella, 2024 | Co‐Designing a Palliative Dementia Care Framework to Support Holistic Assessment and Decision Making: The EMBED‐Care Framework | Journal of the American Medical Informatics Association, | Michigan, Chicago, Ilinois, USA | Implemented in outpatient oncology (GI/lung) and nephrology clinics at Northwestern Medicine (in-person and telehealth) | Total sample: 157 adults receiving care at Northwestern Medicine  Advanced cancer cohort (n = 66):Confirmed stage 4 gastrointestinal malignancy with ≥ 3 months of intravenous chemotherapy, or Confirmed stage 3C/4 lung cancer with first- or second-line chemotherapy for ≥ 3 months Focus-group participants (n = 72): stratified by disease cohort (cancer vs CKD) and role (patients, care partners, healthcare professionals) who provided structured feedback on dashboard mock-ups Chronic kidney disease cohort (n = 91): Confirmed CKD stage ≥ 3, defined by clinical diagnosis or estimated GFR < 60 mL/min/1.73 m²  Eligibility criteria: At least two “dashboard-eligible” visits (including telehealth during COVID-19) with a participating clinician Completion of pre-visit PRO assessments 72 h before visits Co-design contributors (n = 20): core study investigators, patients, care partners, healthcare professionals, and health-IT professionals | To assess the use of a co-designed patient-reported outcome (PRO) clinical dashboard and estimate its impact on shared decision-making (SDM) and symptomatology in adults with advanced cancer or CKD. | 1) Does use of the co-designed PRO dashboard improve SDM? 2) Does it increase self-efficacy in managing treatments and symptoms? 3) Does it improve health-related quality of life (HRQoL)? | Mixed methods Qualitative: Co‐design + focus groups for iterative dashboard refinement Quantitative: Single‐group pretest–posttest (baseline, 3 mo, 6 mo); collaboRATE & PROMIS outcomes; paired t-tests, SRM (α = 0.05) | June 2020 to January 2022 | Shared decision-making model by Elwyn et al.; Mixed-methods participatory–quantitative hybrid design  Guided by Dartmouth’s Coproduction Design and Implementation Flow Model (CDIFM) for stakeholder co-design | |
| Chaudry, 2022 | Successful Aging for Community-Dwelling Older Adults: An Experimental Study with a Tablet App. | International Journal of Environmental Research and Public Health | South Bend, Indiana,United States. | The participants were from rural, low-income independent living facilities. | Age Range: 56 to 83 years (mean age = 65 years) Gender Distribution: 14 females and 11 males Ethnicity: 22 participants identified as African Americans, 2 as White Caucasians, and 1 as Multi-Ethnic Socioeconomic Status: 19 participants reported an income of less than $10,000 per year Education: 15 participants had between 13-15 years of formal education, while 10 had 12 years or less Living Situation: All but five participants lived alone Technology Experience: 17 participants rated themselves as novice users of technology, while 8 felt comfortable using tablets due to prior experience | To test the feasibility of the eSeniorCare app in improving self-perceptions of successful aging among community-dwelling older adults with low socioeconomic status. | RQ1: Does the eSeniorCare app improve self-management behaviors? RQ2: How does the app impact health-related quality of life (HRQoL)? RQ3: What are the usability barriers and benefits as perceived by participants? | Mixed-methods quantitative surveys, (objective measurement and Qualitative interviews ( subjective, user-centered perspectives). | 24 week study period | Single-arm, pre–post mixed-methods feasibility study  Iterative, human-centered design over five years to build and refine the eSeniorCare tablet app in collaboration with target users and care staff | |
| Chen, 2021 | An Intelligent Individualized Cardiovascular App for Risk Elimination (iCARE) for Individuals With Coronary Heart Disease: Development and Usability Testing Analysis | JMIR MHEALTH AND UHEALTH | Beijing, China | Inpatient and ambulatory cardiology services at two university-affiliated hospitals in Beijing | Stage I (January–March 2019): 88 hospitalized CHD patients (72% male [63/88]; mean age 60.0 years, SD 9.9) who reported at least one unhealthy behavior completed task analyses and the first Health-ITUES survey   Stage II (June 2020): 61 CHD patients (85% male [52/61]; mean age 53.0 years, SD 8.2) randomized to the intervention arm of the ongoing RCT and who had used the iCARE app for at least six months completed a second Health-ITUES evaluation | To describe the development of the iCARE system and to evaluate its usability among individuals with CHD | Not explicitly stated; implicitly: (1) How can iCARE be developed via a user-centered design? (2) What is the usability of iCARE—measured by satisfaction, usefulness, and ease of use—among CHD patients? | Mixed-methods Quantitative: Task metrics (time, errors, completion rates) Health-ITUES survey (20 Likert items; stats: means, SD, t-tests, Mann–Whitney U, χ²) App-use logs (frequency, feature use over 6 mo) Qualitative: Expert heuristic inspections (ISO 9241-11) Structured debriefs with users (usability problems) | January 2019 to June 2020 | User-Centered Design (UCD): patient/provider needs drive feature design Intervention Mapping: systematic, evidence-based development of “IF–THEN” behavior-change algorithms Contemplation–Action–Maintenance (CAM) Model: theoretical basis for app feedback loops Nursing Process Framework: assessment → planning → implementation → evaluation of clinical modules | |
| Daniels, 2023 | Promoting physical activity and a healthy active lifestyle in community-dwelling older adults: a design thinking approach for the development of a mobile health application | Frontiers in Public Health | Hasselt, Belgium | Participants were community-dwelling older adults | Age Range: 65 years and older  Gender Distribution: Predominantly female (91%)   Other Characteristics: Participants were community-dwelling older adults with no severe illness, good understanding of the Dutch language, and the ability to actively participate in interviews, workshops, and pilot testing. | To identify barriers and facilitators to physical activity (PA) in older adults, develop an mHealth app promoting PA and an active healthy lifestyle, and test it | RQ1: What are the barriers and facilitators to PA in older adults? RQ2 How can an mHealth app be developed to promote PA and a healthy lifestyle in older adults? RQ3: What is the effectiveness of the developed mHealth app in promoting PA among older adults? | Mixed methods approach combining qualitative, in-depth interviews and co-creation workshops with quantitative, usability scales and user experience questionnaires. | Not specified | The behaviour change wheel (BCW) helps develop interventions by identifying targeted behaviours and their barriers and facilitators. Human-centred design thinking process encompassing empathy, ideation, prototyping, and testing phases  Self Determination theory (SDT) emphasises empathy, ideation, and iterative prototyping to develop solutions that address the needs of the end user | |
| Davies, 2024 | Shared decision-making and disease management in advanced cancer and chronic kidney disease using patient-  reported outcome dashboards | *Health Expectations* | London, United Kingdom | Community groups (usual meeting places) & care homes; workshops held in person and via Zoom | People with dementia Involved via two small DEEP workshops and a PPI panel PPI group: 3 people with mild dementia (including younger-onset cases); guided development, reviewed prototypes in two PPI workshops and a final user-testing workshop Family carers (n = 18) Median age 63.5 years (range 42–81) Predominantly women (16/18) All identified as White (English or other) Educational attainment: 10/18 held a degree or equivalent Davies-2024- Co‐Designi… Health & social care practitioners (n = 55)  Median age 47 years (range 28–66)  Mostly women (49/55)  Ethnicity: White 45, Black 3, Asian 2, Other 2  Educational attainment: 43/55 held a degree or equivalent  Roles spanned multidisciplinary dementia care:  28 nursing specialists  4 care-home managers/leads  2 general practitioners  Digital-health leads  15 allied roles (e.g. occupational therapists, speech & language therapists, dementia support officers) | To co-design a digital app-based palliative dementia care framework for holistic needs assessment and decision-making with people with dementia, family carers and professionals | 1. How should the EMBED-Care Framework integrate holistic assessment and decision support? 2. What app features and implementation requirements do stakeholders identify? 3. How usable and acceptable is the co-designed prototype? | qualitative iterative co-design workshops, think-aloud user-testing sessions and stakeholder feedback exercises - analysed via framework analysis of evidence syntheses and thematic analysis of workshop outputs and user feedback, with iterative logic‐model refinement, rather than through statistical hypothesis testing or quantitative surveys. | Not specified | A systematic, iterative partnership co-design methodology, situated within the Medical Research Council’s guidance on developing complex interventions and framed by a logic model derived from earlier EMBED-Care evidence syntheses. | |
| Doyle, 2021 | A Digital Platform to Support Self-management of Multiple Chronic Conditions (ProACT): Findings in Relation to Engagement During a One-Year Proof-of-Concept Trial. | JOURNAL OF MEDICAL INTERNET RESEARCH | Ireland and Belgium | The participants were older adults with multimorbidity who lived at home and self-managed their conditions with the help of the ProACT digital health platform and their care network. | Age range: >65 years (n=119), 60 years. Gender distribution: n=120 (F=66, M=54). Other relevant characteristics: Participants diagnosed with two or more chronic conditions: diabetes, chronic obstructive pulmonary disease (COPD), chronic heart disease (CHD), congestive heart failure (CHF). Most common condition combination: diabetes and CHD (n=44). Mean number of conditions per participant: 2.5 (SD=0.7). Average comorbidities: 6.8 (SD=3.4)." | to design and develop a digital health platform, ProACT, for facilitating older adults self-managing multimorbidity, with support from their care network, and evaluate end user engagement and experiences with this platform through a 12-month trial. | RQ1: How do older adults with multimorbidity engage with the ProACT platform over a year-long trial? RQ2: What are the benefits and challenges of using the ProACT platform for older adults with multimorbidity and their care network? RQ3: How usable and burdensome is the ProACT platform for older adults with multimorbidity? RQ4:How does the ProACT platform impact the self-management behaviors and outcomes of older adults with multimorbidity? | Mixed-methods: Quantitative: Interactions with the platform, usability and user burden questionnaires, and sensor and well-being data. Qualitative: Semistructured interviews with participants and thematic analysis of their experiences. | 12-month period | Action Research–Based Proof-of-Concept Trial with Four iterative research cycles evaluated at time points T1–T4 Mixed-Methods Evaluation Combining qualitative insights and quantitative metrics to assess engagement and usability User-Centered, Iterative Design with End users (older adults, caregivers, healthcare professionals) | |
| Hawley-Hague, 2020 | Smartphone Apps to Support Falls Rehabilitation Exercise: App Development and Usability and Acceptability Study. | JMIR MHEALTH AND UHEALTH | United Kingdom (University of Manchester and associated partners). | The participants in the study were recruited from a community fall rehabilitation service in Manchester. The setting included patients' homes, where the health care professionals delivered the exercise program and conducted the usability testing[ | Older Adults/Patients: PPI Workshops (Design): 8 participants, all over 60 years old, all White British, with 6 females. Usability (Implementation): 7 participants, mean age 77.1 years (SD 8.53, range 64-92), with 4 White British, 2 White Irish, and 1 Indian. There were 4 men. Health Professionals: PPI Workshops (Design): 5 participants, all White British, with 4 females. Professional backgrounds included 2 physiotherapists, 1 occupational therapist, and 2 rehabilitation assistants. Usability (Implementation): 11 participants, all White British, with 8 females. Professional backgrounds included 9 physiotherapists, 1 nurse, and 1 occupational therapist. | To develop motivational smartphone apps co-designed with health professionals and older adults to support exercise adherence and to evaluate the apps' usability and acceptability | RQ1: How effective are the smartphone apps in supporting older adults to perform strength and balance exercises? RQ2: Are the apps usable and acceptable to both health professionals and older adults? RQ3: What are the barriers and facilitators to using the apps? | The study is mixed-methods, combining qualitative (interviews, focus groups, participatory evaluation) and quantitative (usability testing, issue logs) approaches to assess the apps' usability and acceptability. | Not specified | Design Framework: System Development Life Cycle with three iterative phases (analysis, design, implementation)  Mixed‐methods Approach, guided by the MRC framework for complex interventions  Human‐Centered Design and Patient & Public Involvement throughout development | |
| Hilberger, 2025 | Design of a Mobile App and a Clinical Trial Management System for Cognitive Health and Dementia Risk Reduction: User‑Centered Design Approach | JMIR Aging | Graz, Austria (lead site); multicenter across Austria, Finland, Sweden, Italy. | Development of digital intervention tools within the European LETHE dementia prevention project; user involvement via workshops and feedback sessions; evaluated in a 4-country randomized trial setting. | *Older adults:* 156 participants at risk of cognitive decline (mean ~69 years, ~65% female; sites in AT/FIN/SWE/IT) – provided mobile app usability feedback. *Professionals:* 21 health professionals (clinicians/research staff) – provided feedback on the CTMS. | To design and implement a user‑centered digital platform (a mobile app for older participants and a clinical trial management system for clinicians) to support a multidomain lifestyle intervention aimed at reducing dementia risk in older adults. | RQ1. How can a user‑centred design approach be used to develop a mobile app and clinical trial management system suitable for older adults at risk of cognitive decline and clinical staff?  RQ2. What is the usability and acceptability of the LETHE mobile app among older adult participants?  RQ3. What is the usability and acceptability of the clinical trial management system from the perspective of health professionals? | Mixed‑methods with qualitative participatory design workshops; and quantitative usability testing (SUS) | 2 years (ongoing RCT; interim usability data at 1 month post-baseline) | User‑Centered Design (UCD): requirement analysis workshops (with older adults and clinicians), creation of user personas, iterative design (wireframes, clickable prototypes) with continuous feedback and advisory board input, initial usability testing (think‑aloud tasks), and refinement of the LETHE app and CTMS; followed by usability evaluation (System Usability Scale surveys for app users, and structured user feedback surveys for CTMS | |
| Hoffman, 2020 | Development and Field Testing of a Long-Term Care Decision Aid Website for Older Adults: Engaging Patients and Caregivers in User-Centered Design | The Gerontologist, | New England, US | An Aging Resource Center that serves approximately 1,600 people per year from remote rural New England towns (e.g., 100 to 10,000 people)1. | Age Range: Primarily 65-84 years old. Gender Distribution: Majority female (83.3%). Ethnicity: Mostly White/Caucasian (91.7%). Comfort with Technology: Varying levels of experience with computers. Financial Means: Most had social security (83.3%) and other financial means. Caregiving Status: Six participants were caregivers. Care Needs: Considering long-term care services within the next year. | To develop a web-based decision aid to help older adults and their families make informed and personalized decisions about long-term care and financing options | RQ1:Can engaging older adults and their family members in codesign produce a long-term care decision aid website that is useful, feasible, and acceptable? RQ2: How does the decision aid website affect the knowledge, decisional conflict, preferences, and action planning of older adults and their families? RQ3: What are the needs and preferences of older adults and their families when using a decision aid website? | Mixed-methods study.  Quantitative data from questionnaires to assess participants’ knowledge, decisional conflict, acceptability, and usage of the decision aid website. Qualitative data from cognitive interviews, semistructured interviews, and ThinkAloud sessions to elicit participants’ feedback, suggestions, and experiences with the decision aid website. | Not specified | Mixed-methods, user-centered design guided by the Ottawa Decision Support Framework and IPDAS standards, integrating qualitative codesign with quantitative field testing .  Sequential three‐phase process (1) codesign and initial prototype; (2) iterative paper storyboard testing; (3) live Internet field test | |
| Nambisan, 2022 | A Comprehensive Digital Self-care Support System for Older Adults With Multiple Chronic Conditions: Development, Feasibility, and Usability Testing of myHESTIA | Journal of Applied Gerontology | Wisconsin, USA | Senior centers (Dept. on Aging dining sites), online community recruitment, home-based system use | Age range: ≥60 years (mean age: ~66 years). Gender distribution: Mixed (Phase 3: 66.7% male, 33.3% female). Literacy: Participants able to read and write in Arabic.All cognitively intact for Phase 1; various levels of cognitive, functional, and physical impairments in later phases. | To evaluate the need for a comprehensive digital self-care support system (CDSSS) for older adults with multiple chronic conditions (MCC) and to examine whether such a system can be developed to enable daily capture of self-care data | RQ1:Can a Comprehensive Digital Self-care Support System (CDSSS) be designed for older adults with multiple chronic conditions (MCC)?  RQ2: Will the CDSSS enable the daily capture of self-care data from older adults? | Multiphase, mixed methods design guided by Creswell and Plano Clark (2011).  Phase-1: Qualitative interviews and agile system development for user needs assessment and prototype development. Phase-2: Quantitative survey to assess the usefulness of the CDSSS and preliminary user evaluation of the prototype. Phase-3: Mixed methods: 4-week small group usability and feasibility testing of the tracking component of the prototype. | Three phase study with the final usabilty and feasibility testing phase of 4 weeks | | User-centered, agile prototype development   Theoretical grounding in self-management (Holman & Lorig; Ryan & Sawin) |
| Sein, 2024 | Tailored Self-Management App to Support Older Adults With Cancer and Multimorbidity: Development and Usability Testing | JMIR Aging | British Columbia, Canada | Community-based recruitment and remote/in-person usability testing with older adults and caregivers | Total Participants:18; Age Range: 40–88 years (most between 70–75). Older Adults: 15, 8 participated in design of low fidelity prototypes, 10 evaluated the medium fidelity prototype, 2 older adults participated in both of those workshops. Caregivers:2 participated in low fidelity design, 1 particitaed in medium fidelity evaluation. Gender Distribution: 12/18 (67%) were women and 6/18 (33%) were men. | To report on the user evaluations of a co-designed self- and symptom-management app (Mantra) tailored to support older adults living with cancer and multimorbidit | RQ1: What are the usability perceptions of older adults and caregivers regarding the Mantra app? RQ2: Can older adults complete key self-management tasks using the app? RQ3: How can the app be integrated into existing health management routines for older adults with cancer and multimorbidity? | Mixed Methods, using iterative co-design and usability testing. The study followed the Design Thinking model (Empathize, Define, Ideate, Prototype, Test) and incorporated Grey’s Self-Management Theory. | Multiphase design and evaluationcConducted between 2021 and 2023 (24 months | Design Thinking framework to guide the iterative process. User-Centred Design (UCD): brainwriting, conceptual modeling, codesign of low-fidelity prototype and usability testing of medium fidelity prototype. Grey’s Revised Self-Management Theory to align app features with self-management outcomes. Mixed-methods evaluation: - Quantitative: Task completion rates and System Usability Scale (SUS) - Qualitative: Thematic analysis of post-evaluation interviews using a six-stage coding approach Tools: Axure, Sketch, and Figma for prototyping; RITE (Rapid Iterative Testing and Evaluation) method for usability refinement.  Let me know if you'd like this added to your comparative matrix or formatted for export. | |
| Villa-Garcia, 2022 | The development of a platform to ensure an integrated care plan for older adults with complex care needs living at home | Journal of Integrated Care | Catalonia, Spain. | Tested in a home care company where older adults received care services. Participants were selected from different centers within the public health and social system of Catalonia. | Older Adults7 older adults in focus groups, 50 older adults in field testing. Age Range: Primarily 65 years and older. Informal Caregivers Participants: 6 informal caregivers in interviews. Professional Caregivers and Healthcare/Social Workers Participants: 57 professional caregivers in focus groups, 12 experts in a modified Delphi consensus, 7 social workers in field testing. | To design and develop a digital platform to support the creation and monitoring of care plans tailored to older adults with complex care needs who are users of a home care service. | RQ1:How can user-centered design (UCD) principles be employed to create an effective platform for integrated care? RQ2: What are the key functionalities and requirements for such a platform based on stakeholder input? RQ3: How feasible is the implementation of this digital platform in real-world home care settings? | Mixed-methods case study, Qualitative interviews, focus groups, Delphi consensus to explore user needs and feedback, and quantitative usability testing, structured task completion metrics to evaluate the platform's usability and feasibility in real world settings. | Not specified | Medical Research Council framework for developing complex interventions, User-Centered Design (UCD) and Agile principles | |

### Results Extracted

| **Author (year), Intervention** | **Core Design Approach** | **Methods** | **Tools** | **Theoretical Frameworks** | **Who is the dashboard for** | **Dashboard related features** |
| --- | --- | --- | --- | --- | --- | --- |
| Abujarad et al. (2021), VOICES Tool | User-Centred Design (UCD) | Focus groups (n=3) were held to test and validate the concept of elder mistreatment electronic screening that included older adults, caregivers, healthcare professionals, and social workers (n=31).  Usability evaluation study with a representative sample (n = 14) of older adults was conducted in a simulated ED setting consisting of four tasks. They collected qualitative and quantitative data from the participants using surveys, audio- and video-recordings, and talk-aloud methods. | System Usability Scale (SUS) was used to measure the usability of the VOICES tool, a validated 10-item questionnaire that assesses the ease of use and satisfaction of a system. Computer efficacy scale (CES) measured the comfortability with technology of the participants,atool that assesses the confidence and self-efficacy of using computers. International Positive and Negative Affect Schedule Short Form (I-PANAS-SF): was used to measure the emotional reactions of the participants to the VOICES tool, a validated 10-item survey that assesses the positive and negative affective states of a person. | Theories of planned behavior & self-determination | Clinician dashboard interface (under development at the time of the study) | Summarised report generation Visual and textual flagging of responses suggestive of mistreatment  Secure access for confidential review by healthcare providers in emergency or clinical settings. Tailored alerts to support clinical decision-making based on participant inputs. |
| Afolabi et al. (2025), Medication Decision Support Tool | Human‑Centred Design (HCD), stakeholder co‑design | Iterative co‑design using stakeholder committee meetings with pharmacists, clinicians, and researchers; facilitated discussions to identify requirements, refine decision logic, and iteratively develop a digital prototype. Qualitative synthesis of stakeholder input informed tool structure and content. | Prototype digital decision support interface; workshop facilitation materials; structured meeting documentation. | Human‑Centred Design principles; shared decision‑making concepts (implicitly applied). | Healthcare professionals involved in medication prescribing and review for older adults. | Rule‑based medication decision logic Structured decision pathways supporting polypharmacy review Summarised patient‑specific recommendations for clinical use |
| Bao et al. (2025), SingaporeWALK physical activity app | Participatory co‑design; Human‑Centred Design | Participatory design workshops with older adults to elicit needs and preferences; iterative prototyping and refinement; pre–post feasibility evaluation combining qualitative feedback with quantitative measures of usability and physical activity engagement. | Mobile app prototype; wearable step trackers; questionnaires and activity logs for usability and engagement assessment. | Fogg Behaviour Model for behaviour change; active ageing principles. | Older adults using the app for self‑monitoring and activity planning; researchers monitoring engagement data. | At‑a‑glance activity summaries (e.g. steps, goals) Visual progress indicators supporting self‑monitoring Backend views for monitoring usage and adherence trends |
| Cella et al. (2024), PRO Dashboard | Iterative, Human-Centered Design | Qualitative co-design: Four iterative phases (brainstorming, workflow mapping, mock-up consensus, pilot usability) with a 20-member core team + 72-participant focus groups using semi-structured guides and session notes   Quantitative evaluation: Single-group pretest–posttest at baseline, 3- and 6-month follow-up; within-participant analyses with paired t-tests, McNemar’s χ², Wilcoxon signed-rank as appropriate   Implementation: 1-month soft launch; automated MyChart® portal alerts ~72 h pre-visit; telephone/in-clinic PRO assistance; weekly multidisciplinary monitoring meetings | PRO instruments: PROMIS short forms & computer-adaptive tests (depression, fatigue, physical function; + anxiety & pain for cancer), collaboRATE SDM scale, CASE self-efficacy, FACT-G7, COST-FACIT financial toxicity  Data platforms: Epic MyChart® portal; REDCap for 3-/6-month surveys; EHR-embedded PRO scoring/dashboard David-2024-Shared decis…  Analysis software & metrics: R v4.1.2; paired-samples t-tests (α = 0.05); standardized response means (SRM); nonparametric tests (McNemar’s χ², Wilcoxon signed-rank) | Successful Aging framework, Goal setting principles | Both healthcare professionals and patients | A live, co-designed PRO clinical dashboard is integrated into the Northwestern Medicine EHR via Epic MyChart® with:  Real-time PRO visualization: Trending graphs of most recent PROMIS domains—depression, fatigue, physical function (plus anxiety and pain for cancer cohort) Symptom & goals section: Five open-ended prompts capturing (1) top concerns for visit, (2) most concerning side effects, (3) treatment goals, (4) personal goals/values, and (5) collaboration preferences Clinical data integration: Automatic pull-in of recent lab values, vital signs, and treatment milestones alongside PROs. Patient portal alerts: Automated notifications ~72 h pre-visit to prompt PRO completion, with phone/clinic assistance for non-responders  Interactive display with Graphs and tables supporting discussion of symptom trajectories and goal alignment. |
| Chaudry et al. (2022), eSeniorCare app | User-Centered Design (4 phases) | Quantitative surveys at Baseline (pre-study) and 24-week follow-up (post-study) SF-12v2 Health Survey (Physical & Mental Component Scores) Older Person’s QoL (OPQoL) – Health dimension only  QunatitaiveAnalysis: Regression-based imputation for missing SF-12 items Paired Wilcoxon signed-rank tests for pre/post comparisons (α=0.05)  Qualitative 45 min semi-structured interviews (benefits, barriers, usability)  Thematic analysis via open coding—three researchers independently coded transcripts, then reconciled themes | Screening: Saint Louis University Mental Status Exam (SLUMSE) for cognitive eligibility Intervention Platform: Android tablets with eSeniorCare app (Java/REST API/MySQL) Web portal (PHP) for staff monitoring Training: Weekly facilitator-led tablet workshops with paper manuals Software: Microsoft Excel for descriptive statistics Stata for imputation and Wilcoxon testing Behavioral Framework: SMART goal-setting within app design | Intervention Mapping framework, Contemplation-Action-Maintenance (CAM) model, Nursing process principles | Lightweight, at-a-glance visual overviews present within the tablet app for older users and on the RLHA (Resident Life and Health Administrator, the AiP staff member who enrolled participants, entered their meds/goals, and monitored their data via the portal ) web portal for AiP(Aging-in-Place) staff | Within the tablet app (for older users) Goal progress “rings”: Each goal is represented by an icon surrounded by a circular progress ring that fills in blue as the user logs progress toward that goal (grey = outstanding, blue = completed percentage) .  On the RLHA web portal (for the AiP staff) Noncompliance “alert list”: Participants who miss medications or goals appear in a simple list of alerts rather than a chart.  Request queue: New goal or medication requests are presented in a tabular list for review. |
| Chen et al. (2021), iCARE | Design Thinking Process | Four-Phase Development Functional design (mind-maps, brainstorming) Iterative prototyping (architecture, UI) Expert heuristic inspections & walkthroughs End-user usability testing (2 stages)  Usability Testing Stage I: task analysis (n = 88 inpatients) Stage II: Health-ITUES survey (n = 61 outpatients, ≥6 mo use)  Data Analysis Descriptive stats (mean, SD, IQR) Inferential tests (Kolmogorov–Smirnov; t-tests; Mann–Whitney U; χ²; α = .05) | Software Development & Release Management Java & Node.js for building the patient- and provider-side apps  MicroService architecture hosted on Linux with containerization via Docker, orchestrated through a JHipster registry  GitLab for version control and managing releases across alpha/beta stages  Test-first practices (acceptance-criteria–driven development) following an agile mindset to ensure code quality and rapid iteration   UI Prototyping & Visualization Axure RP 8 to draft initial interface wireframes and workflows  Flinto 26.0.5 for high-fidelity mock-ups embodying the six user-friendly design principles (structure, simplicity, visibility, feedback, tolerance, reuse)  Custom infographics and charting libraries for in-app risk trajectory and Cardiac Health Score visualizations  Standards & Heuristic Evaluation ISO 9241-11 usability standard to structure task analyses (efficiency, effectiveness, satisfaction) Standardized evaluation forms + timers to record task completion times, error rates, and completeness scores against ISO criteria   End-User Usability Testing Instruments Modified Health-ITUES (20-item Likert survey covering impact, usefulness, ease-of-use, user control) in Chinese, validated for CHD patients (Cronbach α .74–.90)  App-use logs (frequency of access, feature usage over 6 months) captured via the cloud platform to quantify real-world engagement   Statistical Analysis using SPSS v24.0 (IBM) for One-sample Kolmogorov–Smirnov tests for normality Descriptive statistics (means, SDs, IQRs) Inferential tests: t-tests, Mann–Whitney U, χ² (α = .05) | Design Thinking, Behavior Change Wheel (BCW) | A patient-facing dashboard (within the mobile app) and a provider-facing dashboard (via the care-provider app and cloud platform) | Patient Dashboard Home-Page Summary (summarized health report with diagnoses, risk factors, goals) Cardiac Health Score Visualization (dynamic “score” reflecting real-time adherence) Risk Trajectory Charts (projected 10-year risk and plaque progression) Trend Charts & Peer Ranking (behavior/physiology trends + comparative ranking)  Provider Dashboard Patient Data Aggregation (real-time behaviors, vitals, wearable data) Health Report & Goal Review (view/adjust the patient’s summary and goals) Intervention Management (create/edit “IF–THEN” rules and content) Follow-Up Workflows (schedule visits, record follow-ups, track progress) Analytics & Reporting (cohort-level trends, outlier detection, built-in reports) |
| Daniels et al. (2023), mHealth app | Co-design, Dartmouth’s Coproduction Design and Implementation Flow Model (CDIFM) | Recruitment via senior organisations and convenience sampling; domain experts included in 2nd workshop. Empathy and defining phase: Semi-structured interviews (40–85 min, audio-recorded) using a predefined guide  Thematic analysis following Braun & Clarke’s six-step approach Ideation & Prototyping Phase  Two 4-hr co-creative workshops: (1) older adults only (n=16); (2) older adults + experts (n=14). Small-group brainstorming, paper and digital mock-ups for screen design, deductive coding aligned with BCW taxonomy  Testing (Sport Day Event) Think ALoud exploration of prototype on uniform tablets; 1-hr exploration  Structured feedback via: UEQ, SUS and additional survey items | Qualitative Analysis BCW Taxonomy for mapping intervention functions (Michie et al. 2011)  Measurement Instruments UEQ: Assesses attractiveness, perspicuity, efficiency, dependability, stimulation, novelty. SUS: Standard 5-point Likert usability scale Daniels-2023-Promoting-…. App Backend logs: Custom neighborhood calendar, push-notification logs, and usage analytics. | Dartmouth’s Coproduction Design and Implementation Flow Model (CDIFM) | Progress tracking view for older adults and Backend analytics forstudy team/reserachers | A glanceable “progress” screen showing exercise charts and feedback snippets for self-monitoring  Community Calendar with a centralized feed of local activity events acting as a social-activity dashboard  Personalized “home” view summarizing baseline activity, motivation profile, and goals on entry—serves as a proposed dashboard   Backend Analytics Dashboard Internal research-facing dashboard surfacing usage logs, notification metrics, and aggregate progress data |
| Davies et al. (2024), EMBED Car | Systematic & Iterative Co-design | Underpinning evidence synthesis (Stage 1): Framework analysis of systematic and policy reviews, cohort studies and large routine clinical datasets to identify 12 core palliative dementia care domains  Logic‐model development and refinement: Creation of an initial logic model from prior EMBED-Care work, iteratively refined through stakeholder input in workshops  Co-design workshops (Stage 2): Eight workshops (2 in-person; 6 online) supplemented by asynchronous feedback (email, Google Jamboard). Techniques included modified nominal-group processes for consensus, small-group breakouts, and think-aloud tasks to shape Framework content, app features and implementation requirements User-testing sessions (Stage 3): Two final in-person workshops using think-aloud and cognitive-walkthrough methods to evaluate the prototype’s usability and acceptability  Patient and Public Involvement (PPI): Ongoing engagement with a PPI panel (three people with mild dementia, including younger-onset, plus seven family carers), through two dedicated PPI workshops, email review and the final user-testing workshop  Data analysis: Framework analysis of evidence-synthesis outputs and matrix mapping. Inductive thematic analysis of workshop transcripts, Jamboard notes and user-testing observations. Iterative logic-model refinement driven by emerging themes and stakeholder feedback | aTouchAway app & web dashboard for delivering the EMBED-Care prototype  IPOS-Dem holistic needs assessment embedded in the app  Google Jamboard + email for asynchronous idea capture  Think-aloud protocols Usability testing method (“cognitive walking through” the prototype) based on Van Someren et al.’s approach Modified nominal-group technique to prioritise and reach consensus  Iterative logic-model mapping to guide each prototype iteration  Framework Analysis Matrix‐based synthesis of prior evidence reviews and routine data to identify core palliative-care domain | Medical Research Council (MRC) guidance for complex interventions, Person-centred care, comfort, shared decision-making, integrated palliative care | Web-based dashboard professional users (e.g. the senior nurse or care-home manager in charge of coordinating a team; community nursing leads; GPs in primary care) | Currently implemented: Patient/Resident roster view with latest IPOS-Dem scores Colour-coded alert indicators (red/amber flags based on score thresholds)  Trend-tracking displays of symptom-and-concern scores over time  Filtering and prioritisation controls (by alert status, date of last assessment, etc.)  Proposed or under refinement for upcoming trials: Action-logging functionality to record responses/actions taken against alerts (e.g. task assignments, follow-up notes) |
| Doyle et al. (2021), ProACT platform with CareApp | Action Research Design, Iterative Design & Development | Semistructured Interviews Conducted in participants’ homes at each ARC to explore experiences, benefits, and barriers  Validated Questionnaires • System Usability Scale (SUS) • User Burden Questionnaire (six burden constructs)  Usage Analytics Log-based metrics: daily symptom readings; CareApp section visits and duration  Thematic Analysis Inductive coding of interview transcripts in NVivo (Ireland) and MAXQDA (Belgium)  Descriptive Statistics Engagement rates, SUS scores (threshold > 68), burden scores over time | ProACT CareApp (responsive web app): dashboard (“flower”), View Readings, Add Info, Tips, My Goals Off-the-Shelf Sensors: blood pressure cuff, glucometer, pulse oximeter, weight scale, smartwatch SIMS & Triage Dashboard: personalized user management, alert generation and clinical triage interface CABIE+ & KITE Platforms: source-agnostic data brokering (CABIE+), scalable cloud storage and analytics orchestration (KITE) CareAnalytics Modules: Data Cleaner, Goal Recommender, User Engagement Analyser Qualitative Coding Software: NVivo, MAXQDA Accessibility & Usability Frameworks: WCAG 2.0, BBC Mobile Guideline | (Implicitly based on burden of multimorbidity, conflict awareness/resolution) | Older adults, informal and formal carers, and clinical triage dashboard for dediacted triage nurses monitoring large cohorts | CareApp: Person-with-Multimorbidity “Flower” Dashboard with “flower” petals for each tracked parameter, color-coded for: up-to-date/normal, out-of-range, or missing readings View Readings Trends Time‐series charts of vitals and well-being metrics, with simple zoom controls “My Goals” Dashboard with weekly progress bars for activity goals (steps, distance, minutes), plus recommendations from the Goal Recommender Care-Network Dashboards (CareApps for carers & healthcare professionals) Read-only views of the person’s flower status, trend charts, and alert notifications (permission‐based sharing) Clinical Triage Dashboard (SIMS-Triage) Prioritized alert list (red/yellow/green), status tags (New/Under Review/Resolved), and one-click drill-downs into individual trend data The “Probabilistic Health Profile” and “Adaptive Education Panel” were proposed but not yet deployed during the trial. |
| Hawley-Hague et al. (2020), "My Activity Programme" and "Motivate Me" apps | Co-designed, Human-Centered Design (HCD) | Codesign Workshops: PPI sessions with older adults and health professionals (prototype feedback)  Usability Testing: 3-week field trial using Samsung Galaxy S4 devices; issue logs & field notes  Qualitative Evaluation: Semistructured interviews with patients; focus groups with professionals  Framework analysis in NVivo 11 (double coding for rigour) | Software & Devices: NVivo 11; Samsung Galaxy S4 smartphones with 4G/SIM cards  Behavioral Frameworks: Behavior Change Technique taxonomy; Theory of Planned Behavior; Technology Acceptance Model  Prototyping: Wireframes, storyboards, iterative app prototypes  Standard Materials: Later Life Training exercise icons/booklets for interface consistency | Behavior Change Techniques (BCTs), Theory of Planned Behavior (TPB), Self-Determination Theory (SDT), Medical Research Council (MRC) framework, Technology Acceptance Model (TAM) | Lightweight, at-a-glance visual summary present in "Motivate Me" for healthcare professionals and "My Activity Programme" for patients | Motivate Me (for healthcare professionals): The “home” view lists each patient’s programmed exercises, their self-reported completion status, and any messages sent. This list-style summary lets professionals monitor adherence at a glance, send feedback, and adjust goals. My Activity Programme (for patients): The main screen shows that day’s scheduled exercises, lets the user tap to log each one, and surfaces incoming motivational pop-ups. It functions as a personal “dashboard” by always displaying the current program and any outstanding tasks. |
| Hilberger et al. (2025), LETHE app and clinical trial management system | User‑Centred Design (UCD); iterative requirements‑driven development | Requirements analysis workshops with older adults and professionals; iterative design and prototyping; structured usability testing using validated questionnaires; refinement of both participant‑facing app and professional dashboards. | LETHE mobile application; clinical trial management system (CTMS); System Usability Scale (SUS); structured usability surveys. | User‑Centred Design principles; human–computer interaction (HCI) concepts. | Older adults participating in clinical trials; researchers and clinicians managing trial data. | Participant overview dashboards summarising engagement and task completion Trial‑level monitoring views for researchers Structured displays supporting study coordination and oversight |
| Hoffman et al. (2020), Web-based long-term care decision aid | User-Centered Design | Phase 1: Needs Assessment & Prototype Development Scoping review of long‐term care literature and environmental scan of existing aids Focus groups, surveys, and stakeholder advisory panel to elicit user requirements Creation of personas and paper mock-ups for initial design   Phase 2: Storyboarding & Cognitive Interviews Three iterative cycles of paper-prototype review with healthcare professionals and caregivers (n = 2–3 per round) In-depth cognitive interviews with older adults to refine wording, layout, and navigation   Phase 3: Field Testing Think-Aloud usability sessions with 12 older adults (individuals and couples) Pre-/post-questionnaires assessing: Knowledge (5-item quiz) Decisional conflict via the 4-item SURE Scale Acceptability via the Ottawa Acceptability Scale (9 items) | Data Collection & Recording: Think-Aloud protocol; Morae screen‐capture software for session recording   Measurement Instruments: SURE Decisional Conflict Scale (1 = Yes/0 = No; score ≤ 3 indicates conflict)  Ottawa Acceptability Scale (percentage positive ratings on each item)  Development Platform: WordPress v2015 for website coding and easy updates   Design Artifacts: Design Strategies Map and paper storyboards to guide iterative refinements | Decision science, cognitive science, human computer interaction, health services research, Ottawa Decision Support Framework | Primary Users: Older adults considering long-term care options Family caregivers (including both local and long-distance)  Secondary Users: Aging resource center staff and healthcare advisors | Side-by-side comparison chart where users can select and compare multiple long-term care options visually "My Decision Summary" page Automatically generated, personalized printout summarizing user inputs (knowledge, preferences, gaps) integrating input data into an actionable format Interactive input fields (values clarification, self-quizzes) where users enter preferences and receive feedback and tailored content The website works like a simple patient-facing dashboard with comparisons, personalized summaries, and interactive steps—but without complex visuals or real-time data. |
| Nambisan et al. (2022), myHESTIA (Comprehensive Digital Self-Care Support System - CDSSS) | Iterative UCD, Agile Design, MRC guidance | Phase 1(Qualitative): Semi-structured interviews (N=10 older adults + 10 caregivers); literature review; reflexive thematic analysis  Phase 2(Quantitative): Prototype demo; 5-point Likert surveys (older adults N=25; healthcare professionals N=15); follow-up interviews; descriptive statistics + qualitative coding Phase 3(mixed Methods): 4-week single-arm usability trial (N=10); daily app-usage logs; Online Community Experience Scale; open feedback; means/SDs + thematic synthesis | myHESTIA app: condition-specific trackers, sense-making dashboards, gamified correlation, journaling, community forum  Qualtrics for online surveys   PROMIS patient-reported outcome scales for tracker calibration   Braun & Clarke’s six-step reflexive thematic analysis framework   Online Community Experience Scale (22-item semantic differential) | Medical Research Council guidance for complex interventions | Primary Users: Community-dwelling older adults with multiple chronic conditions  Secondary Users: Informal caregivers and geriatric healthcare professionals (physicians, nurses, trainees) who can be granted view or edit access to the dashboard | Tabular reports presenting raw daily tracker entries in spreadsheet style Simple line/bar charts displaying user-friendly trends over time  Gamified “play with my data” tool for exploring statistical correlations between conditions Auto-customized dashboard elements based on the user’s selected chronic conditions Shared access for caregivers and healthcare professionals to view or edit user data |
| Sien et al. (2024), Mantra App | Design Thinking + User-Centered Design (UCD) | Qualitative: - Brainwriting exercises with older adults and caregivers (n=10) to identify and prioritize self-management tasks - Semi-structured interviews during usability evaluations (n=10) to explore integration into daily routines and app acceptability - Thematic analysis of interview transcripts using a six-stage coding framework (familiarization, coding, theme development, refinement)  Quantitative: - System Usability Scale (SUS) administered to all medium-fidelity prototype evaluators (n=10); average score: 87 (“best imaginable” usability) - Descriptive statistics used to summarize: - eHealth literacy (eHEALS): mean = 30.6, SD = 9.0, range = 8–40 - Frailty status (VES-13): only one participant classified as frail - Participant demographics and comorbidity profiles - Task completion tracking across core app features (daily reports, weekly summaries, reminders); 90% of participants completed all tasks | System Usability Scale (SUS):10-item questionnaire used to assess ease of use, satisfaction, and learnability of the Mantra app.  Vulnerable Elders Survey (VES-13): to assess frailty risk among older adults, based on age, self-rated health, and functional limitations. eHealth Literacy Scale (eHEALS): to measure participants’ confidence in locating, evaluating, and applying electronic health information. Prototyping Platforms: Axure and Sketch were used to develop low-fidelity wireframes; Figma was used to create medium-fidelity mock-ups with interactive features and refined aesthetics. Usability Testing Method: Online via Zoom and in person using Rapid Iterative Testing and Evaluation (RITE) which is effective in identifying and resolving usability issues quickly through real-time participant feedback and iterative design adjustments. | Grey’s Revised Self-Management Theory Design Thinking Framework Human-Computer Interaction (HCI) Principles for Older Adults | Primary users: Older adults with cancer and multimorbidity Secondary users: Informal caregivers and healthcare providers via shareable summaries and reports | Calendar-style symptom and mood tracking Weekly visual summaries of symptom trends Color-coded indicators for symptom severity Shareable reports for caregivers and providers Tabs for navigating reports, summaries, and education content Autosave functionality for partially completed entries |
| Villa-Garcia et al. (2022), Integrated Care Platform | Multiphase, Mixed Methods, Agile Method | Qualitative:  Focus groups with older adults (n=7) and professional caregivers (n=7)  Semi-structured interviews with informal caregivers (n=6)  Modified Delphi consensus with health/social care experts (n=12)  Iterative Prototyping & Evaluation:  Low-fidelity wireframes → high-fidelity Figma mock-ups  Laboratory usability testing (3 participants per stakeholder group)  12-week field trial with 7 social workers and 50 older adults | Design & Prototyping: Figma for wireframes and interactive mock-ups  Development Process: Scrum (weekly sprints, cross-functional driving team)  Consensus Building: Delphi rounds and co-creation workshops  Usability Testing: Task-based lab scripts, issue-logging templates, digital-literacy guides | (PROMIS scales for tracker development) | Chiefly for social workers and, by extension, other care-team members | Summary overview of the person’s clinical, functional, cognitive, mental-health and social needs (the “most important aspects of the person”). Care-plan snapshot showing current objectives, upcoming activities or visits (an agenda view). Visual alerts for key issues (e.g. unmet goals or flagged assessment items). Care-team roster, clarifying roles/permissions and who’s responsible for each element of the plan. |
