# Supplementary material for: Human-centred design of digital health dashboards in care of older adults: a scoping review
Source: BMJ Open. 2026 Jul 17;16(7):e113525. doi: 10.1136/bmjopen-2025-113525 (PMC13384139; doi:10.1136/bmjopen-2025-113525)
Supplement: online supplemental appendix 4 [file bmjopen-16-7-s004.docx]

## Appendix 4: Quality appraisal of studies

### A: Summarised quality appraisal Checklist

#### Quality appraisal of Mixed Methods Studies using the MMAT.

| **Study** | **Criteria from the Mixed Methods Appraisal Tool** | | | | | | | | | | | | | | | | | | | | | | | | | **Overall score (%)** |
| --- | --- | --- | --- | --- | --- | --- | --- | --- | --- | --- | --- | --- | --- | --- | --- | --- | --- | --- | --- | --- | --- | --- | --- | --- | --- | --- |
|  | 1.1 | 1.2 | 1.3 | 1.4 | 1.5 | 2.1 | 2.2 | 2.3 | 2.4 | 2.5 | 3.1 | 3.2 | 3.3 | 3.4 | 3.5 | 4.1 | 4.2 | 4.3 | 4.4 | 4.5 | 5.1 | 5.2 | 5.3 | 5.4 | 5.5 |  |
| Abujarad (2021) | Y | Y | Y | Y | Y |  |  |  |  |  |  |  |  |  |  | Y | N | Y | U | Y | Y | Y | Y | U | Y | 82.3 |
| Bao (2025) | Y | Y | Y | Y | Y |  |  |  |  |  |  |  |  |  |  | Y | N | Y | U | Y | Y | Y | Y | U | Y | 82.3 |
| Cella (2024) | Y | Y | Y | Y | Y |  |  |  |  |  | N | Y | Y | U | Y | Y | N | Y | U | Y | Y | Y | Y | U | Y | 77.2 |
| Chaudhry (2022) | Y | Y | Y | Y | Y |  |  |  |  |  | N | Y | Y | U | Y | Y | N | Y | U | Y | Y | Y | Y | U | Y | 77.2 |
| Chen (2021) | Y | Y | Y | Y | Y |  |  |  |  |  | N | Y | Y | U | Y | Y | N | Y | U | Y | Y | Y | Y | U | Y | 77.2 |
| Daniels (2023) | Y | Y | Y | Y | Y |  |  |  |  |  | N | Y | Y | U | Y | Y | N | Y | U | Y | Y | Y | Y | U | Y | 77.2 |
| Doyle (2021) | Y | Y | Y | Y | Y |  |  |  |  |  | N | Y | Y | U | Y | Y | N | Y | U | Y | Y | Y | Y | U | Y | 77.2 |
| Hawley-Hague (2020) | Y | Y | Y | Y | Y |  |  |  |  |  | N | Y | Y | U | Y | Y | N | Y | U | Y | Y | Y | Y | U | Y | 77.2 |
| Hilberger (2025) | Y | Y | Y | Y | Y |  |  |  |  |  | N | Y | Y | U | Y | Y | N | Y | U | Y | Y | Y | Y | U | Y | 82.3 |
| Hoffman (2019) | Y | Y | Y | Y | Y |  |  |  |  |  | N | Y | Y | U | Y | Y | N | Y | U | Y | Y | Y | Y | U | Y | 77.2 |
| Nambisan (2023) | Y | Y | Y | Y | Y |  |  |  |  |  | N | Y | Y | U | Y | Y | N | Y | U | Y | Y | Y | Y | U | Y | 77.2 |
| Sien (2024) | Y | Y | Y | Y | Y |  |  |  |  |  | N | Y | Y | U | Y | Y | N | Y | U | Y | Y | Y | Y | U | Y | 77.2 |
| Villa-Garcia (2022) | Y | Y | Y | Y | Y |  |  |  |  |  | N | Y | Y | U | Y | Y | N | Y | U | Y | Y | Y | Y | U | Y | 77.2 |

Y=yes, criterion met; N=no criterion not met; U=unclear or cannot tell whether criterion met. The overall score is the percentage of criteria met. For mixed methods studies where multiple domains were assessed, the overall quality score is the lowest score of the study components.

The full MMAT can be found here: <http://mixedmethodsappraisaltoolpublic.pbworks.com/w/file/fetch/127916259/MMAT_2018_criteria-manual_2018-08-01_ENG.pdf>

#### Quality appraisal of Qualitative study using the JBI Checklist.

| **Study** | **Criteria from the JBI Checklist** | | | | | | | | | | **Overall score (%)** |
| --- | --- | --- | --- | --- | --- | --- | --- | --- | --- | --- | --- |
|  | 1 | 2 | 3 | 4 | 5 | 6 | 7 | 8 | 9 | 10 |  |
| Afolabi (2025) | U | Y | Y | Y | Y | N | U | N | Y | Y | 60 |
| Davies (2024) | U | Y | Y | Y | Y | Y | U | Y | Y | Y | 80 |

Y=yes, criterion met; N=no criterion not met; U=unclear or cannot tell whether criterion met. The overall score is the percentage of criteria met. For mixed methods studies where multiple domains were assessed, the overall quality score is the lowest score of the study components .

The full JBI Checkilst can be found here: <http://mixedmethodsappraisaltoolpublic.pbworks.com/w/file/fetch/127916259/MMAT_2018_criteria-manual_2018-08-01_ENG.pdf>

## B: Detailed Quality Appraisal of Included Studies

### Mixed Methods studies

#### Study 1: Abujarad

**Part I: Mixed Methods Appraisal Tool (MMAT), version 2018**

| **Category of study designs** | **Methodological quality criteria** | **Responses** | | | |
| --- | --- | --- | --- | --- | --- |
|  |  | Yes | No | Can’t tell | Comments |
| Screening questions  (for all types) | S1. Are there clear research questions? | X |  |  | To develop and evaluate the usability of the VOICES |
|  | S2. Do the collected data allow to address the research questions? | X |  |  | qualitative (focus groups) and quantitative (usability testing) data address the research questions. |
|  | *Further appraisal may not be feasible or appropriate when the answer is ‘No’ or ‘Can’t tell’ to one or both screening questions.* | | | | |
| 1. Qualitative | 1.1. Is the qualitative approach appropriate to answer the research question? | X |  |  | Focus groups, open-ended discussions, surveys, and feedback, |
|  | 1.2. Are the qualitative data collection methods adequate to address the research question? | X |  |  |  |
|  | 1.3. Are the findings adequately derived from the data? | X |  |  | participant feedback and thematic analysis. |
|  | 1.4. Is the interpretation of results sufficiently substantiated by data? | X |  |  | participant quotes and survey results |
|  | 1.5. Is there coherence between qualitative data sources, collection, analysis and interpretation? | X |  |  |  |
| 2. Quantitative randomized controlled trials | 2.1. Is randomization appropriately performed? |  |  |  | *Not applicable: No RCT was conducted.* |
|  | 2.2. Are the groups comparable at baseline? |  |  |  |  |
|  | 2.3. Are there complete outcome data? |  |  |  |  |
|  | 2.4. Are outcome assessors blinded to the intervention provided? |  |  |  |  |
|  | 2.5 Did the participants adhere to the assigned intervention? |  |  |  |  |
| 3. Quantitative non-randomized | 3.1. Are the participants representative of the target population? |  |  |  | *Not applicable: No non-randomized intervention.* |
|  | 3.2. Are measurements appropriate regarding both the outcome and intervention (or exposure)? |  |  |  |  |
|  | 3.3. Are there complete outcome data? |  |  |  |  |
|  | 3.4. Are the confounders accounted for in the design and analysis? |  |  |  |  |
|  | 3.5. During the study period, is the intervention administered (or exposure occurred) as intended? |  |  |  |  |
| 4. Quantitative descriptive | 4.1. Is the sampling strategy relevant to address the research question? | X |  |  | purposively sampled |
|  | 4.2. Is the sample representative of the target population? |  | X |  | small and limited to cognitively intact, English-speaking older adults in one region. |
|  | 4.3. Are the measurements appropriate? | X |  |  | Standardized tools (e.g., SUS, I-PANAS-SF) and surveys were used. |
|  | 4.4. Is the risk of nonresponse bias low? |  |  | X | response rates are not reported. |
|  | 4.5. Is the statistical analysis appropriate to answer the research question? | X |  |  | Descriptive statistics and appropriate analyses are reported. |
| 5. Mixed methods | 5.1. Is there an adequate rationale for using a mixed methods design to address the research question? | X |  |  | to capture both usability (quantitative) and user experience (qualitative). |
|  | 5.2. Are the different components of the study effectively integrated to answer the research question? | X |  |  |  |
|  | 5.3. Are the outputs of the integration of qualitative and quantitative components adequately interpreted? | X |  |  |  |
|  | 5.4. Are divergences and inconsistencies between quantitative and qualitative results adequately addressed? |  |  | X | no explicit discussion |
|  | 5.5. Do the different components of the study adhere to the quality criteria of each tradition of the methods involved? | X |  |  |  |

**Result:**

14 out of 17 applicable criteria are met (“Yes”), 1 is “No”, and 2 are “Can’t tell”. This gives an overall MMAT score of 82.3%.

#### Study 2: Bao

**Part I: Mixed Methods Appraisal Tool (MMAT), version 2018**

| **Category of study designs** | **Methodological quality criteria** | **Responses** | | | |
| --- | --- | --- | --- | --- | --- |
|  |  | Yes | No | Can’t tell | Comments |
| Screening questions  (for all types) | S1. Are there clear research questions? | X |  |  | To design and evaluate the usability and feasibility of the SGWALK app for active ageing. |
|  | S2. Do the collected data allow to address the research questions? | X |  |  | Qualitative participatory workshops and quantitative pre–post data address both design and evaluation aims.. |
|  | *Further appraisal may not be feasible or appropriate when the answer is ‘No’ or ‘Can’t tell’ to one or both screening questions.* | | | | |
| 1. Qualitative | 1.1. Is the qualitative approach appropriate to answer the research question? | X |  |  | Iterative c-design cycles in participatory design workshops to explore user needs and inform design decisions. |
|  | 1.2. Are the qualitative data collection methods adequate to address the research question? | X |  |  |  |
|  | 1.3. Are the findings adequately derived from the data? | X |  |  | Design insights are clearly drawn from participant input.. |
|  | 1.4. Is the interpretation of results sufficiently substantiated by data? | X |  |  | Interpretation supported through participant feedback and design iterations. |
|  | 1.5. Is there coherence between qualitative data sources, collection, analysis and interpretation? | X |  |  | Clear alignment between workshops, prototype refinement and reported themes. |
| 2. Quantitative randomized controlled trials | 2.1. Is randomization appropriately performed? |  |  |  | *Not applicable: No RCT was conducted.* |
|  | 2.2. Are the groups comparable at baseline? |  |  |  |  |
|  | 2.3. Are there complete outcome data? |  |  |  |  |
|  | 2.4. Are outcome assessors blinded to the intervention provided? |  |  |  |  |
|  | 2.5 Did the participants adhere to the assigned intervention? |  |  |  |  |
| 3. Quantitative non-randomized | 3.1. Are the participants representative of the target population? |  |  |  | *Not applicable: No non-randomized intervention.* |
|  | 3.2. Are measurements appropriate regarding both the outcome and intervention (or exposure)? |  |  |  |  |
|  | 3.3. Are there complete outcome data? |  |  |  |  |
|  | 3.4. Are the confounders accounted for in the design and analysis? |  |  |  |  |
|  | 3.5. During the study period, is the intervention administered (or exposure occurred) as intended? |  |  |  |  |
| 4. Quantitative descriptive | 4.1. Is the sampling strategy relevant to address the research question? | X |  |  | purposively sampled |
|  | 4.2. Is the sample representative of the target population? |  | X |  | Limited to digitally capable older adults in one community setting. |
|  | 4.3. Are the measurements appropriate? | X |  |  | Standardised self‑report measures and device‑generated data were used. |
|  | 4.4. Is the risk of nonresponse bias low? |  |  | X | Attrition and non‑response not fully reported |
|  | 4.5. Is the statistical analysis appropriate to answer the research question? | X |  |  | Descriptive statistics and appropriate analyses are reported. |
| 5. Mixed methods | 5.1. Is there an adequate rationale for using a mixed methods design to address the research question? | X |  |  | To integrate participatory design insights with usability and feasibility outcomes |
|  | 5.2. Are the different components of the study effectively integrated to answer the research question? | X |  |  | Qualitative insights inform design and interpretation of outcomes*.* |
|  | 5.3. Are the outputs of the integration of qualitative and quantitative components adequately interpreted? | X |  |  | Joint interpretation of design and usage findings is coherent. |
|  | 5.4. Are divergences and inconsistencies between quantitative and qualitative results adequately addressed? |  |  | X | no explicit discussion |
|  | 5.5. Do the different components of the study adhere to the quality criteria of each tradition of the methods involved? | X |  |  | Both qualitative and quantitative components meet feasibility‑study standards. |

**Result:**

14 out of 17 applicable criteria are met (“Yes”), 1 is “No”, and 2 are “Can’t tell”. This gives an overall MMAT score of 82.3%.

#### Study 3: Cella (2024)

**Part I: Mixed Methods Appraisal Tool (MMAT), version 2018**

| **Category of study designs** | **Methodological quality criteria** | **Responses** | | | |  |
| --- | --- | --- | --- | --- | --- | --- |
|  |  | Yes | No | Can’t tell | Comments | |
| Screening questions  (for all types) | S1. Are there clear research questions? | X |  |  | assess the impact of a co-designed patient-reported outcome (PRO) dashboard | |
|  | S2. Do the collected data allow to address the research questions? | X |  |  | quantitative (pre-post surveys, clinical outcomes) and qualitative (co-design process, feedback) data | |
|  | *Further appraisal may not be feasible or appropriate when the answer is ‘No’ or ‘Can’t tell’ to one or both screening questions.* | | | | |  |
| 1. Qualitative | 1.1. Is the qualitative approach appropriate to answer the research question? | X |  |  | Semi-structured focus groups and iterative feedback | |
|  | 1.2. Are the qualitative data collection methods adequate to address the research question? | X |  |  |  |  |
|  | 1.3. Are the findings adequately derived from the data? | X |  |  | Dashboard features and priorities were directly informed by participant feedback | |
|  | 1.4. Is the interpretation of results sufficiently substantiated by data? | X |  |  | design choices linked to participant needs | |
|  | 1.5. Is there coherence between qualitative data sources, collection, analysis and interpretation? | X |  |  |  | |
| 2. Quantitative randomized controlled trials | 2.1. Is randomization appropriately performed? |  |  |  | *Not applicable: No RCT was conducted.* | |
|  | 2.2. Are the groups comparable at baseline? |  |  |  |  |  |
|  | 2.3. Are there complete outcome data? |  |  |  |  |  |
|  | 2.4. Are outcome assessors blinded to the intervention provided? |  |  |  |  |  |
|  | 2.5 Did the participants adhere to the assigned intervention? |  |  |  |  |  |
| 3. Quantitative non-randomized | 3.1. Are the participants representative of the target population? |  | X |  | mostly White, younger, and lower proportion of Hispanic/Latino and Black participants | |
|  | 3.2. Are measurements appropriate regarding both the outcome and intervention (or exposure)? | X |  |  | Validated tools (collaboRATE, PROMIS, FACT-G7, etc) | |
|  | 3.3. Are there complete outcome data? | X |  |  | 157/184 participants completed baseline and 3-month follow-up; attrition and missing data are reported | |
|  | 3.4. Are the confounders accounted for in the design and analysis? |  |  | X | acknowledges limitations and potential selection bias but does not adjust for confounders | |
|  | 3.5. During the study period, is the intervention administered (or exposure occurred) as intended? | X |  |  |  | |
| 4. Quantitative descriptive | 4.1. Is the sampling strategy relevant to address the research question? | X |  |  | Purposive sampling | |
|  | 4.2. Is the sample representative of the target population? |  | X |  |  | |
|  | 4.3. Are the measurements appropriate? | X |  |  |  | |
|  | 4.4. Is the risk of nonresponse bias low? |  |  | X | Unclear risk; response rates and differences between enrolled and non-enrolled are reported. | |
|  | 4.5. Is the statistical analysis appropriate to answer the research question? | X |  |  | Paired t-tests, Wilcoxon signed-rank tests, and SRM are appropriate for pre-post design. | |
| 5. Mixed methods | 5.1. Is there an adequate rationale for using a mixed methods design to address the research question? | X |  |  |  | |
|  | 5.2. Are the different components of the study effectively integrated to answer the research question? | X |  |  |  | |
|  | 5.3. Are the outputs of the integration of qualitative and quantitative components adequately interpreted? | X |  |  |  | |
|  | 5.4. Are divergences and inconsistencies between quantitative and qualitative results adequately addressed? |  |  | X | does not explicitly discuss divergences or inconsistencies between data types. | |
|  | 5.5. Do the different components of the study adhere to the quality criteria of each tradition of the methods involved? | X |  |  |  | |

**Results:**

17 out of 22 applicable criteria are met (“Yes”), 2 are “No”, and 3 are “Can’t tell”. This gives an overall MMAT score of **77.2%**.

#### Study 4: Chaudhry (2022)

**Part I: Mixed Methods Appraisal Tool (MMAT), version 2018**

| **Category of study designs** | **Methodological quality criteria** | **Responses** | | | |
| --- | --- | --- | --- | --- | --- |
|  |  | Yes | No | Can’t tell | Comments |
| Screening questions  (for all types) | S1. Are there clear research questions? | X |  |  | test the feasibility of a tablet-based mHealth app |
|  | S2. Do the collected data allow to address the research questions? | X |  |  | qualitative (interviews) and quantitative (pre-post surveys) |
|  | *Further appraisal may not be feasible or appropriate when the answer is ‘No’ or ‘Can’t tell’ to one or both screening questions.* | | | | |
| 1. Qualitative | 1.1. Is the qualitative approach appropriate to answer the research question? | X |  |  | Semi-structured interviews, open coding, and thematic analysis |
|  | 1.2. Are the qualitative data collection methods adequate to address the research question? | X |  |  |  |
|  | 1.3. Are the findings adequately derived from the data? | X |  |  | linked to participant quotes and thematic categories*.* |
|  | 1.4. Is the interpretation of results sufficiently substantiated by data? | X |  |  | direct quotes and survey results. |
|  | 1.5. Is there coherence between qualitative data sources, collection, analysis and interpretation? | X |  |  |  |
| 2. Quantitative randomized controlled trials | 2.1. Is randomization appropriately performed? |  |  |  | *Not applicable: No RCT was conducted.* |
|  | 2.2. Are the groups comparable at baseline? |  |  |  |  |
|  | 2.3. Are there complete outcome data? |  |  |  |  |
|  | 2.4. Are outcome assessors blinded to the intervention provided? |  |  |  |  |
|  | 2.5 Did the participants adhere to the assigned intervention? |  |  |  |  |
| 3. Quantitative non-randomized | 3.1. Are the participants representative of the target population? |  | X |  | *The sample is small, from two facilities,* |
|  | 3.2. Are measurements appropriate regarding both the outcome and intervention (or exposure)? | X |  |  | *Validated tools and usage logs* |
|  | 3.3. Are there complete outcome data? | X |  |  | *25/28 participants completed all post-study questionnaires; attrition rate <25%.* |
|  | 3.4. Are the confounders accounted for in the design and analysis? |  |  | X | *acknowledges limitations but does not adjust for confounders in analysis* |
|  | 3.5. During the study period, is the intervention administered (or exposure occurred) as intended? | X |  |  |  |
| 4. Quantitative descriptive | 4.1. Is the sampling strategy relevant to address the research question? | X |  |  | *Purposive sampling* |
|  | 4.2. Is the sample representative of the target population? |  | X |  | *not fully representative* |
|  | 4.3. Are the measurements appropriate? | X |  |  | *Standardized and validated tools* |
|  | 4.4. Is the risk of nonresponse bias low? |  |  | X | *risk is unclear; response rates are not fully reported.* |
|  | 4.5. Is the statistical analysis appropriate to answer the research question? | X |  |  | *Non-parametric tests (Wilcoxon) and thematic analysis* |
| 5. Mixed methods | 5.1. Is there an adequate rationale for using a mixed methods design to address the research question? | X |  |  | *quantitative outcomes and qualitative experiences.* |
|  | 5.2. Are the different components of the study effectively integrated to answer the research question? | X |  |  |  |
|  | 5.3. Are the outputs of the integration of qualitative and quantitative components adequately interpreted? | X |  |  |  |
|  | 5.4. Are divergences and inconsistencies between quantitative and qualitative results adequately addressed? |  |  | X | *does not explicitly discuss divergences or inconsistencies between data types.* |
|  | 5.5. Do the different components of the study adhere to the quality criteria of each tradition of the methods involved? | X |  |  |  |

**Result:**

17 out of 22 applicable criteria are met (“Yes”), 2 are “No”, and 3 are “Can’t tell”. This gives an overall MMAT score of **77.2%**.

#### Study 5: Chen (2021)

**Part I: Mixed Methods Appraisal Tool (MMAT), version 2018**

| **Category of study designs** | **Methodological quality criteria** | **Responses** | | | |
| --- | --- | --- | --- | --- | --- |
|  |  | Yes | No | Can’t tell | Comments |
| Screening questions  (for all types) | S1. Are there clear research questions? | X |  |  | develop and test the usability of the iCARE app |
|  | S2. Do the collected data allow to address the research questions? | X |  |  | qualitative (task analysis, feedback) and quantitative (Health-ITUES survey) data |
|  | *Further appraisal may not be feasible or appropriate when the answer is ‘No’ or ‘Can’t tell’ to one or both screening questions.* | | | | |
| 1. Qualitative | 1.1. Is the qualitative approach appropriate to answer the research question? | X |  |  | Task analysis, usability problem identification, and severity ratings |
|  | 1.2. Are the qualitative data collection methods adequate to address the research question? | X |  |  |  |
|  | 1.3. Are the findings adequately derived from the data? | X |  |  | *Usability problems and user feedback linked to design changes and findings* |
|  | 1.4. Is the interpretation of results sufficiently substantiated by data? | X |  |  | *direct feedback and usability scores, linked to design improvements* |
|  | 1.5. Is there coherence between qualitative data sources, collection, analysis and interpretation? | X |  |  |  |
| 2. Quantitative randomized controlled trials | 2.1. Is randomization appropriately performed? |  |  |  | *No RCT results reported in this paper (RCT protocol mentioned for future work).* |
|  | 2.2. Are the groups comparable at baseline? |  |  |  |  |
|  | 2.3. Are there complete outcome data? |  |  |  |  |
|  | 2.4. Are outcome assessors blinded to the intervention provided? |  |  |  |  |
|  | 2.5 Did the participants adhere to the assigned intervention? |  |  |  |  |
| 3. Quantitative non-randomized | 3.1. Are the participants representative of the target population? |  | X |  | *mostly male, from two hospitals in Beijing, China* |
|  | 3.2. Are measurements appropriate regarding both the outcome and intervention (or exposure)? | X |  |  | *Validated tools (Health-ITUES) and task analysis* |
|  | 3.3. Are there complete outcome data? | X |  |  | *Usability data collected from all enrolled participants; attrition and refusals reported* |
|  | 3.4. Are the confounders accounted for in the design and analysis? |  |  | X | *knowledges limitations and potential selection bias but does not adjust for confounders* |
|  | 3.5. During the study period, is the intervention administered (or exposure occurred) as intended? | X |  |  |  |
| 4. Quantitative descriptive | 4.1. Is the sampling strategy relevant to address the research question? | X |  |  | *Purposive sampling* |
|  | 4.2. Is the sample representative of the target population? |  | X |  |  |
|  | 4.3. Are the measurements appropriate? | X |  |  |  |
|  | 4.4. Is the risk of nonresponse bias low? |  |  | X | *The risk is unclear; response rates and differences between enrolled and non-enrolled are reported* |
|  | 4.5. Is the statistical analysis appropriate to answer the research question? | X |  |  | *Mann–Whitney U test, chi-square test, and descriptive statistics* |
| 5. Mixed methods | 5.1. Is there an adequate rationale for using a mixed methods design to address the research question? | X |  |  | *to capture both quantitative usability scores and qualitative user feedback* |
|  | 5.2. Are the different components of the study effectively integrated to answer the research question? | X |  |  |  |
|  | 5.3. Are the outputs of the integration of qualitative and quantitative components adequately interpreted? | X |  |  |  |
|  | 5.4. Are divergences and inconsistencies between quantitative and qualitative results adequately addressed? |  |  | X | *does not explicitly discuss divergences or inconsistencies between data types* |
|  | 5.5. Do the different components of the study adhere to the quality criteria of each tradition of the methods involved? | X |  |  |  |

**Results:**

17 out of 22 applicable criteria are met (“Yes”), 2 are “No”, and 3 are “Can’t tell”. This gives an overall MMAT score of **77.2%**.

#### Study 6: Daniels (2023)

**Part I: Mixed Methods Appraisal Tool (MMAT), version 2018**

| **Category of study designs** | **Methodological quality criteria** | **Responses** | | | |
| --- | --- | --- | --- | --- | --- |
|  |  | Yes | No | Can’t tell | Comments |
| Screening questions  (for all types) | S1. Are there clear research questions? | X |  |  | identify barriers/facilitators to physical activity in older adults, develop an mHealth app using design thinking, and test it |
|  | S2. Do the collected data allow to address the research questions? | X |  |  | Qualitative (interviews, workshops) and quantitative (usability scales) data |
|  | *Further appraisal may not be feasible or appropriate when the answer is ‘No’ or ‘Can’t tell’ to one or both screening questions.* | | | | |
| 1. Qualitative | 1.1. Is the qualitative approach appropriate to answer the research question? | X |  |  | Semi-structured interviews, thematic analysis, and participatory workshops |
|  | 1.2. Are the qualitative data collection methods adequate to address the research question? | X |  |  |  |
|  | 1.3. Are the findings adequately derived from the data? | X |  |  | *Themes and app features are clearly linked to participant input and data analysis* |
|  | 1.4. Is the interpretation of results sufficiently substantiated by data? | X |  |  | *direct quotes and feedback, linked to design choices and findings* |
|  | 1.5. Is there coherence between qualitative data sources, collection, analysis and interpretation? | X |  |  |  |
| 2. Quantitative randomized controlled trials | 2.1. Is randomization appropriately performed? |  |  |  | *No RCT was conducted; prototype testing was exploratory.* |
|  | 2.2. Are the groups comparable at baseline? |  |  |  |  |
|  | 2.3. Are there complete outcome data? |  |  |  |  |
|  | 2.4. Are outcome assessors blinded to the intervention provided? |  |  |  |  |
|  | 2.5 Did the participants adhere to the assigned intervention? |  |  |  |  |
| 3. Quantitative non-randomized | 3.1. Are the participants representative of the target population? |  | X |  | *overrepresentation of women, mostly tech-familiar, and motivated participants* |
|  | 3.2. Are measurements appropriate regarding both the outcome and intervention (or exposure)? | X |  |  | *Validated tools (System Usability Scale, User Experience Questionnaire) and structured surveys* |
|  | 3.3. Are there complete outcome data? | X |  |  | *Usability data collected from enrolled participants; attrition and refusals are reported* |
|  | 3.4. Are the confounders accounted for in the design and analysis? |  |  | X | *acknowledges limitations and potential selection bias but does not adjust for confounders* |
|  | 3.5. During the study period, is the intervention administered (or exposure occurred) as intended? | X |  |  |  |
| 4. Quantitative descriptive | 4.1. Is the sampling strategy relevant to address the research question? | X |  |  | *Convenience sampling of older adults in community settings* |
|  | 4.2. Is the sample representative of the target population? |  | X |  |  |
|  | 4.3. Are the measurements appropriate? | X |  |  |  |
|  | 4.4. Is the risk of nonresponse bias low? |  |  | X |  |
|  | 4.5. Is the statistical analysis appropriate to answer the research question? | X |  |  | *Descriptive statistics and appropriate usability metrics* |
| 5. Mixed methods | 5.1. Is there an adequate rationale for using a mixed methods design to address the research question? | X |  |  | *to capture both qualitative insights and quantitative usability scores* |
|  | 5.2. Are the different components of the study effectively integrated to answer the research question? | X |  |  |  |
|  | 5.3. Are the outputs of the integration of qualitative and quantitative components adequately interpreted? | X |  |  |  |
|  | 5.4. Are divergences and inconsistencies between quantitative and qualitative results adequately addressed? |  |  | X | *does not explicitly discuss divergences or inconsistencies between data types* |
|  | 5.5. Do the different components of the study adhere to the quality criteria of each tradition of the methods involved? | X |  |  |  |

**Results:**

17 out of 22 applicable criteria are met (“Yes”), 2 are “No”, and 3 are “Can’t tell”. This gives an overall MMAT score of 77.2**%**.

#### Study 7: Doyle (2021)

**Part I: Mixed Methods Appraisal Tool (MMAT), version 2018**

| **Category of study designs** | **Methodological quality criteria** | **Responses** | | | |
| --- | --- | --- | --- | --- | --- |
|  |  | Yes | No | Can’t tell | Comments |
| Screening questions  (for all types) | S1. Are there clear research questions? | X |  |  | to design and evaluate the ProACT digital health platform |
|  | S2. Do the collected data allow to address the research questions? | X |  |  | qualitative (interviews) and quantitative (usability scales, engagement logs) data |
|  | *Further appraisal may not be feasible or appropriate when the answer is ‘No’ or ‘Can’t tell’ to one or both screening questions.* | | | | |
| 1. Qualitative | 1.1. Is the qualitative approach appropriate to answer the research question? | X |  |  | Semistructured interviews and thematic analysis of transcriptions |
|  | 1.2. Are the qualitative data collection methods adequate to address the research question? | X |  |  |  |
|  | 1.3. Are the findings adequately derived from the data? | X |  |  | *Themes and subthemes are clearly linked to participant quotes and data analysis* |
|  | 1.4. Is the interpretation of results sufficiently substantiated by data? | X |  |  | *direct quotes and feedback, linking them to design choices and findings* |
|  | 1.5. Is there coherence between qualitative data sources, collection, analysis and interpretation? | X |  |  |  |
| 2. Quantitative randomized controlled trials | 2.1. Is randomization appropriately performed? |  |  |  | *No RCT was conducted; this was a proof-of-concept trial.* |
|  | 2.2. Are the groups comparable at baseline? |  |  |  |  |
|  | 2.3. Are there complete outcome data? |  |  |  |  |
|  | 2.4. Are outcome assessors blinded to the intervention provided? |  |  |  |  |
|  | 2.5 Did the participants adhere to the assigned intervention? |  |  |  |  |
| 3. Quantitative non-randomized | 3.1. Are the participants representative of the target population? |  | X |  | *mostly male, recruited from specific regions, and motivated participants* |
|  | 3.2. Are measurements appropriate regarding both the outcome and intervention (or exposure)? | X |  |  | *Validated tools (System Usability Scale, user burden questionnaire) and engagement logs* |
|  | 3.3. Are there complete outcome data? | X |  |  | *Usability and engagement data were collected from all enrolled participants; attrition and withdrawals are reported* |
|  | 3.4. Are the confounders accounted for in the design and analysis? |  |  | X | *acknowledges limitations and potential selection bias but does not adjust for confounders* |
|  | 3.5. During the study period, is the intervention administered (or exposure occurred) as intended? | X |  |  |  |
| 4. Quantitative descriptive | 4.1. Is the sampling strategy relevant to address the research question? | X |  |  | *Recruitment of older adults with multimorbidity in Ireland and Belgium* |
|  | 4.2. Is the sample representative of the target population? |  | X |  |  |
|  | 4.3. Are the measurements appropriate? | X |  |  |  |
|  | 4.4. Is the risk of nonresponse bias low? |  |  | X | *risk is unclear; response rates and differences between enrolled and non-enrolled are reported* |
|  | 4.5. Is the statistical analysis appropriate to answer the research question? | X |  |  | *Descriptive statistics and appropriate usability metrics are used* |
| 5. Mixed methods | 5.1. Is there an adequate rationale for using a mixed methods design to address the research question? | X |  |  | *o capture both qualitative insights and quantitative usability/engagement data* |
|  | 5.2. Are the different components of the study effectively integrated to answer the research question? | X |  |  |  |
|  | 5.3. Are the outputs of the integration of qualitative and quantitative components adequately interpreted? | X |  |  |  |
|  | 5.4. Are divergences and inconsistencies between quantitative and qualitative results adequately addressed? |  |  | X | *does not explicitly discuss divergences or inconsistencies between data types* |
|  | 5.5. Do the different components of the study adhere to the quality criteria of each tradition of the methods involved? | X |  |  |  |

**Results:**

17 out of 22 applicable criteria are met (“Yes”), 2 are “No”, and 3 are “Can’t tell”. This gives an overall MMAT score of 77.2**%**.

#### Study 8: Hawley-Hague (2020)

**Part I: Mixed Methods Appraisal Tool (MMAT), version 2018**

| **Category of study designs** | **Methodological quality criteria** | **Responses** | | | |
| --- | --- | --- | --- | --- | --- |
|  |  | Yes | No | Can’t tell | Comments |
| Screening questions  (for all types) | S1. Are there clear research questions? | X |  |  | to develop and evaluate smartphone apps for falls rehabilitation exercise, focusing on usability and acceptability |
|  | S2. Do the collected data allow to address the research questions? | X |  |  | Qualitative (interviews, focus groups, issue logs) and usability data |
|  | *Further appraisal may not be feasible or appropriate when the answer is ‘No’ or ‘Can’t tell’ to one or both screening questions.* | | | | |
| 1. Qualitative | 1.1. Is the qualitative approach appropriate to answer the research question? | X |  |  | Semistructured interviews, focus groups, issue logs, analyzed using framework analysis and participatory evaluation |
|  | 1.2. Are the qualitative data collection methods adequate to address the research question? | X |  |  | Themes and subthemes are clearly linked to participant feedback and data analysis |
|  | 1.3. Are the findings adequately derived from the data? | X |  |  | *direct quotes and feedback, linked to design choices and finding* |
|  | 1.4. Is the interpretation of results sufficiently substantiated by data? | X |  |  |  |
|  | 1.5. Is there coherence between qualitative data sources, collection, analysis and interpretation? | X |  |  |  |
| 2. Quantitative randomized controlled trials | 2.1. Is randomization appropriately performed? |  |  |  | *No RCT was conducted; this was a usability/acceptability study.* |
|  | 2.2. Are the groups comparable at baseline? |  |  |  |  |
|  | 2.3. Are there complete outcome data? |  |  |  |  |
|  | 2.4. Are outcome assessors blinded to the intervention provided? |  |  |  |  |
|  | 2.5 Did the participants adhere to the assigned intervention? |  |  |  |  |
| 3. Quantitative non-randomized | 3.1. Are the participants representative of the target population? |  | X |  | *small sample, mostly volunteers, limited diversity, and short testing period* |
|  | 3.2. Are measurements appropriate regarding both the outcome and intervention (or exposure)? | X |  |  | *Issue logs, usability feedback, and qualitative methods* |
|  | 3.3. Are there complete outcome data? | X |  |  | *Usability and acceptability data were collected from all enrolled participants; attrition and refusals are reported* |
|  | 3.4. Are the confounders accounted for in the design and analysis? |  |  | X | *cknowledges limitations and potential selection bias but does not adjust for confounders* |
|  | 3.5. During the study period, is the intervention administered (or exposure occurred) as intended? | X |  |  |  |
| 4. Quantitative descriptive | 4.1. Is the sampling strategy relevant to address the research question? | X |  |  | *Recruitment of older adults at risk of falls and health professionals* |
|  | 4.2. Is the sample representative of the target population? |  | X |  |  |
|  | 4.3. Are the measurements appropriate? | X |  |  | *Standardized qualitative and usability methods* |
|  | 4.4. Is the risk of nonresponse bias low? |  |  | X | *risk is unclear; response rates and differences between enrolled and non-enrolled are reported* |
|  | 4.5. Is the statistical analysis appropriate to answer the research question? | X |  |  | *Descriptive statistics and appropriate qualitative analysis* |
| 5. Mixed methods | 5.1. Is there an adequate rationale for using a mixed methods design to address the research question? | X |  |  | *to capture both qualitative insights and usability data* |
|  | 5.2. Are the different components of the study effectively integrated to answer the research question? | X |  |  |  |
|  | 5.3. Are the outputs of the integration of qualitative and quantitative components adequately interpreted? | X |  |  |  |
|  | 5.4. Are divergences and inconsistencies between quantitative and qualitative results adequately addressed? |  |  | X | *does not explicitly discuss divergences or inconsistencies between data types* |
|  | 5.5. Do the different components of the study adhere to the quality criteria of each tradition of the methods involved? | X |  |  |  |

**Results:**

17 out of 22 applicable criteria are met (“Yes”), 2 are “No”, and 3 are “Can’t tell”. This gives an overall MMAT score of 77.2**%**.

#### Study 9: Hilberger

**Part I: Mixed Methods Appraisal Tool (MMAT), version 2018**

| **Category of study designs** | **Methodological quality criteria** | **Responses** | | | |
| --- | --- | --- | --- | --- | --- |
|  |  | Yes | No | Can’t tell | Comments |
| Screening questions  (for all types) | S1. Are there clear research questions? | X |  |  | To design and evaluate usability of the LETHE app and clinical trial management system. |
|  | S2. Do the collected data allow to address the research questions? | X |  |  | Qualitative workshops and quantitative usability surveys |
|  | *Further appraisal may not be feasible or appropriate when the answer is ‘No’ or ‘Can’t tell’ to one or both screening questions.* | | | | |
| 1. Qualitative | 1.1. Is the qualitative approach appropriate to answer the research question? | X |  |  | Requirement analysis and workshops suitable for early design. |
|  | 1.2. Are the qualitative data collection methods adequate to address the research question? | X |  |  |  |
|  | 1.3. Are the findings adequately derived from the data? | X |  |  | Design requirements explicitly grounded in stakeholder input. |
|  | 1.4. Is the interpretation of results sufficiently substantiated by data? | X |  |  | Findings consistent with reported workshop outcomes. |
|  | 1.5. Is there coherence between qualitative data sources, collection, analysis and interpretation? | X |  |  |  |
| 2. Quantitative randomized controlled trials | 2.1. Is randomization appropriately performed? |  |  |  | *Not applicable: No RCT was conducted.* |
|  | 2.2. Are the groups comparable at baseline? |  |  |  |  |
|  | 2.3. Are there complete outcome data? |  |  |  |  |
|  | 2.4. Are outcome assessors blinded to the intervention provided? |  |  |  |  |
|  | 2.5 Did the participants adhere to the assigned intervention? |  |  |  |  |
| 3. Quantitative non-randomized | 3.1. Are the participants representative of the target population? |  |  |  | *Not applicable: No non-randomized intervention.* |
|  | 3.2. Are measurements appropriate regarding both the outcome and intervention (or exposure)? |  |  |  |  |
|  | 3.3. Are there complete outcome data? |  |  |  |  |
|  | 3.4. Are the confounders accounted for in the design and analysis? |  |  |  |  |
|  | 3.5. During the study period, is the intervention administered (or exposure occurred) as intended? |  |  |  |  |
| 4. Quantitative descriptive | 4.1. Is the sampling strategy relevant to address the research question? | X |  |  | Targeted recruitment of participants and professionals. |
|  | 4.2. Is the sample representative of the target population? |  | X |  | Participation required baseline digital literacy and trial involvement. |
|  | 4.3. Are the measurements appropriate? | X |  |  | SUS and structured surveys used appropriately. |
|  | 4.4. Is the risk of nonresponse bias low? |  |  | X | Partial survey response rates reported. |
|  | 4.5. Is the statistical analysis appropriate to answer the research question? | X |  |  | Descriptive analysis aligned with usability aims. |
| 5. Mixed methods | 5.1. Is there an adequate rationale for using a mixed methods design to address the research question? | X |  |  | To combine UCD insights with usability metrics. |
|  | 5.2. Are the different components of the study effectively integrated to answer the research question? | X |  |  | Qualitative findings inform system refinement. |
|  | 5.3. Are the outputs of the integration of qualitative and quantitative components adequately interpreted? | X |  |  | Integration supports conclusions on usability and system fit. |
|  | 5.4. Are divergences and inconsistencies between quantitative and qualitative results adequately addressed? |  |  | X | no explicit discussion |
|  | 5.5. Do the different components of the study adhere to the quality criteria of each tradition of the methods involved? | X |  |  | Both traditions implemented with appropriate rigour. |

**Result:**

14 out of 17 applicable criteria are met (“Yes”), 1 is “No”, and 2 are “Can’t tell”. This gives an overall MMAT score of 82.3%.

#### Study 10: Hoffman (2019)

**Part I: Mixed Methods Appraisal Tool (MMAT), version 2018**

| **Category of study designs** | **Methodological quality criteria** | **Responses** | | | |
| --- | --- | --- | --- | --- | --- |
|  |  | Yes | No | Can’t tell | Comments |
| Screening questions  (for all types) | S1. Are there clear research questions? | X |  |  | to develop and field test a long-term care decision aid website for older adults, focusing on user-centered design, feasibility, utility, and acceptability |
|  | S2. Do the collected data allow to address the research questions? | X |  |  | Qualitative (interviews, cognitive walkthroughs) and quantitative (knowledge, decisional conflict, acceptability ratings) data |
|  | *Further appraisal may not be feasible or appropriate when the answer is ‘No’ or ‘Can’t tell’ to one or both screening questions.* | | | | |
| 1. Qualitative | 1.1. Is the qualitative approach appropriate to answer the research question? | X |  |  | Cognitive interviews, think-aloud protocols, open-ended feedback, storyboarding, and participatory design, analyzed for convergent themes |
|  | 1.2. Are the qualitative data collection methods adequate to address the research question? | X |  |  |  |
|  | 1.3. Are the findings adequately derived from the data? | X |  |  |  |
|  | 1.4. Is the interpretation of results sufficiently substantiated by data? | X |  |  |  |
|  | 1.5. Is there coherence between qualitative data sources, collection, analysis and interpretation? | X |  |  |  |
| 2. Quantitative randomized controlled trials | 2.1. Is randomization appropriately performed? |  |  |  | *No RCT was conducted; this was a usability/acceptability study.* |
|  | 2.2. Are the groups comparable at baseline? |  |  |  |  |
|  | 2.3. Are there complete outcome data? |  |  |  |  |
|  | 2.4. Are outcome assessors blinded to the intervention provided? |  |  |  |  |
|  | 2.5 Did the participants adhere to the assigned intervention? |  |  |  |  |
| 3. Quantitative non-randomized | 3.1. Are the participants representative of the target population? |  | X |  | *small, mostly rural, limited diversity, and short testing period* |
|  | 3.2. Are measurements appropriate regarding both the outcome and intervention (or exposure)? | X |  |  | *Knowledge, decisional conflict, and acceptability scales* |
|  | 3.3. Are there complete outcome data? | X |  |  | *Usability and acceptability data were collected from all enrolled participants; attrition and refusals are reported* |
|  | 3.4. Are the confounders accounted for in the design and analysis? |  |  | X | *acknowledges limitations and potential selection bias but does not adjust for confounders* |
|  | 3.5. During the study period, is the intervention administered (or exposure occurred) as intended? | X |  |  |  |
| 4. Quantitative descriptive | 4.1. Is the sampling strategy relevant to address the research question? | X |  |  | *Recruitment of older adults and caregivers in rural settings* |
|  | 4.2. Is the sample representative of the target population? |  | X |  |  |
|  | 4.3. Are the measurements appropriate? | X |  |  |  |
|  | 4.4. Is the risk of nonresponse bias low? |  |  | X | *isk is unclear; response rates and differences between enrolled and non-enrolled are reported* |
|  | 4.5. Is the statistical analysis appropriate to answer the research question? | X |  |  | *Descriptive statistics and appropriate qualitative analysis* |
| 5. Mixed methods | 5.1. Is there an adequate rationale for using a mixed methods design to address the research question? | X |  |  | *to capture both qualitative insights and quantitative usability/acceptability data* |
|  | 5.2. Are the different components of the study effectively integrated to answer the research question? | X |  |  |  |
|  | 5.3. Are the outputs of the integration of qualitative and quantitative components adequately interpreted? | X |  |  |  |
|  | 5.4. Are divergences and inconsistencies between quantitative and qualitative results adequately addressed? |  |  | X | *does not explicitly discuss divergences or inconsistencies between data types* |
|  | 5.5. Do the different components of the study adhere to the quality criteria of each tradition of the methods involved? | X |  |  |  |

**Results:**

17 out of 22 applicable criteria are met (“Yes”), 2 are “No”, and 2 are “Can’t tell”. This gives an overall MMAT score of **77.27%**.

#### Study 12: Nambisan (2023)

**Part I: Mixed Methods Appraisal Tool (MMAT), version 2018**

| **Category of study designs** | **Methodological quality criteria** | **Responses** | | | |
| --- | --- | --- | --- | --- | --- |
|  |  | Yes | No | Can’t tell | Comments |
| Screening questions  (for all types) | S1. Are there clear research questions? | X |  |  | to evaluate the need for a comprehensive digital self-care support system, and to test its feasibility and usability |
|  | S2. Do the collected data allow to address the research questions? | X |  |  | Qualitative (interviews), quantitative (surveys), and mixed methods (usability testing) data |
|  | *Further appraisal may not be feasible or appropriate when the answer is ‘No’ or ‘Can’t tell’ to one or both screening questions.* | | | | |
| 1. Qualitative | 1.1. Is the qualitative approach appropriate to answer the research question? | X |  |  | Interviews and reflexive thematic analysis |
|  | 1.2. Are the qualitative data collection methods adequate to address the research question? | X |  |  |  |
|  | 1.3. Are the findings adequately derived from the data? | X |  |  |  |
|  | 1.4. Is the interpretation of results sufficiently substantiated by data? | X |  |  |  |
|  | 1.5. Is there coherence between qualitative data sources, collection, analysis and interpretation? | X |  |  |  |
| 2. Quantitative randomized controlled trials | 2.1. Is randomization appropriately performed? |  |  |  | *No RCT was conducted; this was a feasibility/usability study.* |
|  | 2.2. Are the groups comparable at baseline? |  |  |  |  |
|  | 2.3. Are there complete outcome data? |  |  |  |  |
|  | 2.4. Are outcome assessors blinded to the intervention provided? |  |  |  |  |
|  | 2.5 Did the participants adhere to the assigned intervention? |  |  |  |  |
| 3. Quantitative non-randomized | 3.1. Are the participants representative of the target population? |  | X |  | *small, mostly volunteers, limited diversity, and short testing period* |
|  | 3.2. Are measurements appropriate regarding both the outcome and intervention (or exposure)? | X |  |  | *Surveys, user experience scales, and log data* |
|  | 3.3. Are there complete outcome data? | X |  |  | *Usability and acceptability data were collected from all enrolled participants; attrition and refusals are reported* |
|  | 3.4. Are the confounders accounted for in the design and analysis? |  |  | X | *acknowledges limitations and potential selection bias but does not adjust for confounders* |
|  | 3.5. During the study period, is the intervention administered (or exposure occurred) as intended? | X |  |  |  |
| 4. Quantitative descriptive | 4.1. Is the sampling strategy relevant to address the research question? | X |  |  | *Recruitment of older adults and caregivers in community and online settings* |
|  | 4.2. Is the sample representative of the target population? |  | X |  |  |
|  | 4.3. Are the measurements appropriate? | X |  |  | *Standardized qualitative and usability methods* |
|  | 4.4. Is the risk of nonresponse bias low? |  |  | X | *risk is unclear; response rates and differences between enrolled and non-enrolled are reported* |
|  | 4.5. Is the statistical analysis appropriate to answer the research question? | X |  |  | *Descriptive statistics* |
| 5. Mixed methods | 5.1. Is there an adequate rationale for using a mixed methods design to address the research question? | X |  |  | *to capture both qualitative insights and quantitative usability/acceptability data* |
|  | 5.2. Are the different components of the study effectively integrated to answer the research question? | X |  |  |  |
|  | 5.3. Are the outputs of the integration of qualitative and quantitative components adequately interpreted? | X |  |  |  |
|  | 5.4. Are divergences and inconsistencies between quantitative and qualitative results adequately addressed? |  |  | X | *does not explicitly discuss divergences or inconsistencies between data types* |
|  | 5.5. Do the different components of the study adhere to the quality criteria of each tradition of the methods involved? | X |  |  |  |

**Results:**

17 out of 22 applicable criteria are met (“Yes”), 2 are “No”, and 3 are “Can’t tell”. This gives an overall MMAT score of 77.2**%**.

#### Study 13: Sien (2024)

**Part I: Mixed Methods Appraisal Tool (MMAT), version 2018**

| **Category of study designs** | **Methodological quality criteria** | **Responses** | | | |
| --- | --- | --- | --- | --- | --- |
|  |  | Yes | No | Can’t tell | Comments |
| Screening questions  (for all types) | S1. Are there clear research questions? | X |  |  | usability perceptions, task completion, and integration of the Mantra app |
|  | S2. Do the collected data allow to address the research questions? | X |  |  | qualitative (interviews, thematic analysis) and quantitative (SUS, task completion) |
|  | *Further appraisal may not be feasible or appropriate when the answer is ‘No’ or ‘Can’t tell’ to one or both screening questions.* | | | | |
| 1. Qualitative | 1.1. Is the qualitative approach appropriate to answer the research question? | X |  |  | Thematic analysis of interviews |
|  | 1.2. Are the qualitative data collection methods adequate to address the research question? | X |  |  | Brainwriting, interviews, and observational feedback |
|  | 1.3. Are the findings adequately derived from the data? | X |  |  |  |
|  | 1.4. Is the interpretation of results sufficiently substantiated by data? | X |  |  | *Direct quotes linked to Grey’s Self-Management Theory* |
|  | 1.5. Is there coherence between qualitative data sources, collection, analysis and interpretation? | X |  |  |  |
| 2. Quantitative randomized controlled trials | 2.1. Is randomization appropriately performed? |  |  |  | *No RCT was conducted; a design, usability, and feasibility study.* |
|  | 2.2. Are the groups comparable at baseline? |  |  |  |  |
|  | 2.3. Are there complete outcome data? |  |  |  |  |
|  | 2.4. Are outcome assessors blinded to the intervention provided? |  |  |  |  |
|  | 2.5 Did the participants adhere to the assigned intervention? |  |  |  |  |
| 3. Quantitative non-randomized | 3.1. Are the participants representative of the target population? |  | X |  | *Sample size was small (n=18)* |
|  | 3.2. Are measurements appropriate regarding both the outcome and intervention (or exposure)? | X |  |  | *SUS for usability, eHEALS for health literacy, and VES-13 for frailty* |
|  | 3.3. Are there complete outcome data? | X |  |  | *All participants completed the usability tasks and surveys; no missing data reported.* |
|  | 3.4. Are the confounders accounted for in the design and analysis? |  | X |  |  |
|  | 3.5. During the study period, is the intervention administered (or exposure occurred) as intended? | X |  |  | *The app was used consistently across participants during usability testing, with no deviations reported.* |
| 4. Quantitative descriptive | 4.1. Is the sampling strategy relevant to address the research question? | X |  |  | *Purposeful sampling of older adults with cancer and multi-morbidity* |
|  | 4.2. Is the sample representative of the target population? |  | X |  | *The sample size is small and skewed towards highly educated and tech-comfortable individuals* |
|  | 4.3. Are the measurements appropriate? | X |  |  | *SUS, eHEALS, VES-13* |
|  | 4.4. Is the risk of nonresponse bias low? | X |  |  | *All recruited participants completed the evaluation* |
|  | 4.5. Is the statistical analysis appropriate to answer the research question? | X |  |  | *Descriptive statistics, SUS* |
| 5. Mixed methods | 5.1. Is there an adequate rationale for using a mixed methods design to address the research question? | X |  |  | *to capture both usability metrics and experiential feedback* |
|  | 5.2. Are the different components of the study effectively integrated to answer the research question? | X |  |  |  |
|  | 5.3. Are the outputs of the integration of qualitative and quantitative components adequately interpreted? | X |  |  |  |
|  | 5.4. Are divergences and inconsistencies between quantitative and qualitative results adequately addressed? |  |  | X | *does not explicitly discuss divergences or inconsistencies between data type* |
|  | 5.5. Do the different components of the study adhere to the quality criteria of each tradition of the methods involved? | X |  |  |  |

**Results:**

17 out of 22 applicable criteria are met (“Yes”), 2 are “No”, and 3 are “Can’t tell”. This gives an overall MMAT score of 77.2**%**.

#### Study 14: Villa Garcia (2022)

**Part I: Mixed Methods Appraisal Tool (MMAT), version 2018**

| **Category of study designs** | **Methodological quality criteria** | **Responses** | | | |
| --- | --- | --- | --- | --- | --- |
|  |  | Yes | No | Can’t tell | Comments |
| Screening questions  (for all types) | S1. Are there clear research questions? | X |  |  | to describe the design and development of a digital platform for integrated care plans |
|  | S2. Do the collected data allow to address the research questions? | X |  |  | Qualitative (focus groups, interviews, consensus), usability, and field testing data |
|  | *Further appraisal may not be feasible or appropriate when the answer is ‘No’ or ‘Can’t tell’ to one or both screening questions.* | | | | |
| 1. Qualitative | 1.1. Is the qualitative approach appropriate to answer the research question? | X |  |  | Focus groups, interviews, iterative feedback, and consensus methods analysed for requirements and usability |
|  | 1.2. Are the qualitative data collection methods adequate to address the research question? | X |  |  |  |
|  | 1.3. Are the findings adequately derived from the data? | X |  |  |  |
|  | 1.4. Is the interpretation of results sufficiently substantiated by data? | X |  |  |  |
|  | 1.5. Is there coherence between qualitative data sources, collection, analysis and interpretation? | X |  |  |  |
| 2. Quantitative randomized controlled trials | 2.1. Is randomization appropriately performed? |  |  |  | *No RCT was conducted; design, usability, and feasibility study.* |
|  | 2.2. Are the groups comparable at baseline? |  |  |  |  |
|  | 2.3. Are there complete outcome data? |  |  |  |  |
|  | 2.4. Are outcome assessors blinded to the intervention provided? |  |  |  |  |
|  | 2.5 Did the participants adhere to the assigned intervention? |  |  |  |  |
| 3. Quantitative non-randomized | 3.1. Are the participants representative of the target population? |  | X |  | *small, mostly volunteers, limited diversity, and short testing period* |
|  | 3.2. Are measurements appropriate regarding both the outcome and intervention (or exposure)? | X |  |  | *Usability, feedback, and field testing* |
|  | 3.3. Are there complete outcome data? | X |  |  | *Usability and acceptability data were collected from all enrolled participants; attrition and refusals are reported* |
|  | 3.4. Are the confounders accounted for in the design and analysis? |  |  | X |  |
|  | 3.5. During the study period, is the intervention administered (or exposure occurred) as intended? | X |  |  |  |
| 4. Quantitative descriptive | 4.1. Is the sampling strategy relevant to address the research question? | X |  |  | *acknowledges limitations and potential selection bias but does not adjust for confounders* |
|  | 4.2. Is the sample representative of the target population? |  | X |  | *Recruitment of older adults, caregivers, and professionals in community and company settings* |
|  | 4.3. Are the measurements appropriate? | X |  |  |  |
|  | 4.4. Is the risk of nonresponse bias low? |  |  | X | *risk is unclear; response rates and differences between enrolled and non-enrolled are reported* |
|  | 4.5. Is the statistical analysis appropriate to answer the research question? | X |  |  | *Descriptive statistics* |
| 5. Mixed methods | 5.1. Is there an adequate rationale for using a mixed methods design to address the research question? | X |  |  | *to capture both qualitative insights and usability/acceptability data* |
|  | 5.2. Are the different components of the study effectively integrated to answer the research question? | X |  |  |  |
|  | 5.3. Are the outputs of the integration of qualitative and quantitative components adequately interpreted? | X |  |  |  |
|  | 5.4. Are divergences and inconsistencies between quantitative and qualitative results adequately addressed? |  |  | X | *While the study triangulates SUS, task completion rates and interview feedback, it does not explicitly discuss divergences or inconsistencies between data type* |
|  | 5.5. Do the different components of the study adhere to the quality criteria of each tradition of the methods involved? | X |  |  |  |

**Results:**

17 out of 22 applicable criteria are met (“Yes”), 2 are “No”, and 3 are “Can’t tell”. This gives an overall MMAT score of 77.2**%**.

### Qualitative Study

**JBI Critical Appraisal Checklist for
Qualitative Research**

Reviewer _Syeda Taqya Amna Arslan ___________________ Date__04/05/2026________________

Author _ Afolabi, T. E., Hilmer, S. N., Etherton‑Beer, C., & Kouladjian O’Donnell, L_____ Year__2025_______ Record Number____1_____

|  | Yes | No | Unclear | Not applicable |
| --- | --- | --- | --- | --- |
| 1. Is there congruity between the stated philosophical perspective and the research methodology? | □ | □ |  | □ |
| 1. Is there congruity between the research methodology and the research question or objectives? |  | □ | □ | □ |
| 1. Is there congruity between the research methodology and the methods used to collect data? |  | □ | □ | □ |
| 1. Is there congruity between the research methodology and the representation and analysis of data? |  | □ | □ | □ |
| 1. Is there congruity between the research methodology and the interpretation of results? |  | □ | □ | □ |
| 1. Is there a statement locating the researcher culturally or theoretically? | □ |  | □ | □ |
| 1. Is the influence of the researcher on the research, and vice- versa, addressed? | □ | □ |  | □ |
| 1. Are participants, and their voices, adequately represented? | □ |  | □ | □ |
| 1. Is the research ethical according to current criteria or, for recent studies, and is there evidence of ethical approval by an appropriate body? |  | □ | □ | □ |
| 1. Do the conclusions drawn in the research report flow from the analysis, or interpretation, of the data? |  | □ | □ | □ |

Overall appraisal: Include  Exclude □ Seek further info □

Comments (Including reason for exclusion)

Overall, the study is of good qualitative rigour with the methodology being carried out in congruity with the research aims, ethical standards being adhered to, and results being reported according to participant data. However, there were minor reporting gaps:

No philosophical perspective is explicitly discussed in the study.

There is also limited reflexive detail about the researcher's influence as the researcher's cultural or theoretical positioning is not discussed, and reflexivity is not discussed in detail. The authors have mentioned that participant conversations were only facilitated without leading them towards an idea, but there is no reflection on how any personal biases may have affected data interpretation. There is also no discussion on how engaging in the research may have influenced the researchers or vice versa, showing that the reflexivity criteria was only partially met

**JBI Critical Appraisal Checklist for
Qualitative Research**

Reviewer _Syeda Taqya Amna Arslan ___________________ Date__21/09/2025________________

Author Nathan Davies, Elizabeth L. Sampson, Jesutofunmi Aworinde, Juliet Gillam, Charlotte Kenten, Kirsten Moore, Bethan Phillips, Catherine Harvey, Janet Anderson, Jane Ward, Catherine J. Evans, Clare Ellis‐Smith, EMBED‐Care Team _____________________ Year__2024_______ Record Number____2_____

|  | Yes | No | Unclear | Not applicable |
| --- | --- | --- | --- | --- |
| 1. Is there congruity between the stated philosophical perspective and the research methodology? | □ | □ |  | □ |
| 1. Is there congruity between the research methodology and the research question or objectives? |  | □ | □ | □ |
| 1. Is there congruity between the research methodology and the methods used to collect data? |  | □ | □ | □ |
| 1. Is there congruity between the research methodology and the representation and analysis of data? |  | □ | □ | □ |
| 1. Is there congruity between the research methodology and the interpretation of results? |  | □ | □ | □ |
| 1. Is there a statement locating the researcher culturally or theoretically? |  | □ | □ | □ |
| 1. Is the influence of the researcher on the research, and vice- versa, addressed? | □ | □ |  | □ |
| 1. Are participants, and their voices, adequately represented? |  | □ | □ | □ |
| 1. Is the research ethical according to current criteria or, for recent studies, and is there evidence of ethical approval by an appropriate body? |  | □ | □ | □ |
| 1. Do the conclusions drawn in the research report flow from the analysis, or interpretation, of the data? |  | □ | □ | □ |

Overall appraisal: Include  Exclude □ Seek further info □

Comments (Including reason for exclusion)

Overall, the study is of good qualitative rigour for an early-stage codesign study with the methodology being carried out in congruity with the research aims, ethical standards being adhered to, and results being reported according to participant data. However, there were minor reporting gaps:

No philosophical perspective is explicitly discussed in the study.

There is also limited reflexive detail about the researcher's influence as the researcher's cultural or theoretical positioning is not discussed, and reflexivity is not discussed in detail. Although the authors note that discussions were facilitated to avoid directing stakeholder responses, there is minimal reflection on how any personal biases may have affected data interpretation. There is also no discussion on how engaging in the research may have influenced the researchers or vice versa, showing that the reflexivity criteria was only partially met

In addition, participant voices are primarily summarised rather than illustrated through direct quotations, which may limit transparency of interpretation.
