# Supplementary material for: Human-centred design of digital health dashboards in care of older adults: a scoping review
Source: BMJ Open. 2026 Jul 17;16(7):e113525. doi: 10.1136/bmjopen-2025-113525 (PMC13384139; doi:10.1136/bmjopen-2025-113525)
Supplement: online supplemental table 1 [file bmjopen-16-7-s005.docx]

| **Study** | **Methods** | **Participants:**  **Older adults (gender, age)**  **Healthcare professionals (gender/age if mentioned)**  **Other participants and details** |
| --- | --- | --- |
| Abujarad-2021 | Focus groups | Older adults=24 (4 M, 20 F, 61–80 years)  Healthcare professionals = 2  Caregivers=2  Social Workers = 3 |
|  | Usability study | Older adults=14 (3 M, 11 F, 62–98 years) |
| Afolabi-2025 | Stakeholder Committee meetings | Older adults = 2  Informal carers = 1  Healthcare professionals = 4 (GP, pharmacist, nurse, geriatrician)  Digital health researchers = 2 |
| Bao-2025 | Participatory design & iterative testing (3 cycles) | Older adults = 32 (NR, 69.4 ± 6.1 years) |
|  | Pre‑post intervention (4‑week community trial) | Older adults = 48 (60–85 years) |
| Cella-2024 | Co-design | Stakeholders (investigators, patients, care partners, healthcare professionals, and health IT professionals) =20 |
|  | Evaluation Study | Older adults = 157 (78M, 79F, 61± 13 years) |
|  | Focus group | Constituents stratified by disease cohort (cancer vs CKD) and role (patients, care partners, healthcare professionals) = 72 |
| Chaudry-2022 | Single-Arm Pre-Post pilot study | Older adults = 25 (11 M, 14 F, 56–83 years, 65 ± 7) |
| Chen-2021 | Stage 1 usability testing | Older adults = 88 (25F,63M, 60 ± 9.9 years) |
|  | Stage 2 usability testing | Older adults = 61 (9F,52M, 53 ± 8.2 years) |
| Daniels-2023 | In-depth Interviews | Older adults = 22 (14 M, 8F, 76.05 ± 6.27 years) |
|  | Co-creative WS 1 | Older adults =16 (10 M, 6F, 68.33 ± 2.77 years) |
|  | Co-creative WS 2 | Older adults = 6 (1 M, 5F, 72.33% ±5.73 years)  Experts (including healthcare professionals) = 8 |
|  | Prototype testing | Older adults =65 (9 M, 56 F, 71.16 ± 4.33) |
| Davies-2024 | 12 Co-design workshops | People with dementia = 6, (3 each in workshop 6 and 7 only, age, gender not reported)  Professionals = 55, 6M,49 F, median age (range)= 47 (28–66) – [Practitioner role: Nurses=28, Care home managers and leads= 4, General practitioners=2, Digital lead, Other=15, Missing=3]  Family Carers =18, 2M, 16 F, Median age (range) 63.5 (42–81) years |
|  | PPI Workshops | People with dementia = 3  Family carers = 7 |
| Doyle-2021 | 3 Action research Cycles - Ireland | Older adults = 60, 36 M, 24 F, 74.23 ± 6.4 years)  8 withdrew and 3 died |
|  | 3 Action research cycles- Belgium | Older adults = 60, 43 M, 17 F, 73.61± 6.49 years  16 withdrew |
| Hawley-Hague- 2020 | informal consultation | Older adults=12, 2 M, 10 F, NR*  Healthcare professionals=16 – [9 PTs, 2 rehabilitation assistants, 2 OTs, and 3 nurses]  Experts = Exercise instructors from 3 falls services |
|  | PPI WS 1 and 2 | Older adults = 8,2 M, 6 F, 60+ years  Healthcare professionals = 5 (4 F) – [2 PTs, 1 OT, 1 rehabilitation assistant, and 1 assistant practitioner] |
|  | Usability Testing | Older adults = 7, 4 M, 3 F, mean age 77.1, ± 8.53 years: range 64-92)  Healthcare professionals = 11 (8 F) - [9 PTs, 1 Nurse, 1 OT] |
| Hilberger-2025 | Requirement analysis for the LETHE app | 21 participants including clinicians, behavioural experts and representatives of public involvement in dementia |
|  | First user test | German speaking older adults = 4 (65-85 |
|  | Requirement Analysis workshop for the CTMS | Healthcare professionals = 13 (neurologists, gerontologists, public health experts, professionals in fields of quality of life and dementia |
|  | Usability testing – mobile app (SUS) | Older adults = 156 trial participants (123 responded: Intervention group = 66/78 and Control group = 57/78) |
|  | Usability testing – CTMS (survey) | Healthcare professionals = 21 (12 responded) |
| Hoffman-2020 | Stakeholder advisory panel - Guided the design and conduct of the study. | Older adults =2, NR, 65–84 years  Healthcare professionals = 6 – [3 geriatric psychiatrists, 3 memory care specialists]  Caregivers =2  Experts =6(2 decision scientists, 4 informaticians) |
|  | Story boarding | Older adults = 5 +12, NR, NR  Caregivers = part of the 12 older adults |
|  | Phase 3 – Field test | Older adults =12, 2 M, 10 F, 80 years old (66.7%, min. 61, max 89) – [4 individuals and 4 couples]  Caregivers = 6 of the 12 Older adults |
| Nambisan-2022 | Phase-1 – Needs Assessment | Older adults =10, 3 M, 7 F, 60–80 years  Caregivers = 10 (>60 years old) |
|  | Phase-2 – Preliminary User Evaluation | Older adults = 25, 5 M, 20 F, 60–>80 years  Healthcare professionals =15 |
|  | Phase-3– Usability and feasibility testing | Older adults = 10, NR, 55-80 years |
| Sien-2024 | Phase 1 – Low Fidelity prototype design | Older adults = 8, NR, NR,  Caregivers = 2 |
|  | Phase 2 – Medium fidelity prototype design | Older adults = 9, NR, NR,  Caregivers = 1 |
|  | Overall | Total participants = 18, 3 caregivers, 15 older adults; 6 M, 12 F, age range of all participants = 40–88 years (only age range was collected); ; 50% aged 70–75 |
| Villa-garcia-2022 | Phase 1 – Conceptualisation, analysis and design | Older adults = 7, NR, 31 to >60 years  Healthcare professionals =12 (physicians (4), social workers (3), nurses (2), OT (1), and a pharmacist (1))  Formal caregivers = 7  Informal caregivers =6  Social workers = 3 |
|  | Phase 2 - Usability testing in a laboratory situation | Older adults =3, NR, NR  Formal caregivers = 3  Informal caregivers = 3 |
|  | Phase 4 - Field testing | Older adults =50, NR, NR  Social Workers = 7 |
| *NR: Not Reported; IT= Information Technology; CTMS = Clinical Trial Management System; SUS = System Usability Scale, OT= Occupational Therapists; PT= Physiotherapists. | | |

Table 1 Methods and participant characteristics of each study
