# Supplementary material for: Human-centred design of digital health dashboards in care of older adults: a scoping review
Source: BMJ Open. 2026 Jul 17;16(7):e113525. doi: 10.1136/bmjopen-2025-113525 (PMC13384139; doi:10.1136/bmjopen-2025-113525)
Supplement: online supplemental table 2 [file bmjopen-16-7-s006.docx]

| Author (year), Intervention | Who is the dashboard for | Dashboard- related features |
| --- | --- | --- |
| Abujarad et al. (2021), VOICES Tool | Clinician dashboard interface (under development at the time of the study) | Summarised report generation Visual and textual flagging of responses suggestive of mistreatment  Secure access for confidential review by healthcare providers in emergency or clinical settings. Tailored alerts |
| Afolabi et al. (2025), My Medicine Goals | Older adults, informal carers, and healthcare professionals | Interactive medication list and goal setting interface  Visual summaries of medicines, goals, and risks  Structured, shareable summaries to support consultations and shared decision‑making  Embedded self‑assessment tools to inform treatment discussions |
| Bao et al. (2025), SingaporeWALK (SGWALK) | Older adults; health coaches and facilitators | Personalised user dashboards displaying physical activity, nutrition, and mental well‑being data  Visual progress tracking using charts, icons, and goal indicators  Real‑time feedback from wearable sensor data  Summary views to support reflection, motivation, and guided feedback from coaches |
| Cella et al. (2024), PRO Dashboard | Both healthcare professionals and patients | A live, co-designed PRO clinical dashboard is integrated into the Northwestern Medicine EHR via Epic MyChart® with: Real-time PRO visualisation.  Symptom & goals section Clinical data integration  Patient portal alerts Interactive display with graphs and tables supporting discussion of symptom trajectories and goal alignment. |
| Chaudry et al. (2022), eSeniorCare app | Older users and AiP (Ageing in Place) staff | **Within the tablet app (for older users)** Goal progress “rings” (grey = outstanding, blue = completed percentage).  **On the RLHA (Resident Life and Health Administrator) web portal for the AiP (Ageing-in-Place) staff** Noncompliance “alert list” for missed medications or goals  Tabular lists of new goal or medication requests for review. |
| Chen et al. (2021), iCARE | Older adults and Healthcare providers | **Patient Dashboard (within mobile app)** Home-Page Summary  Dynamic Cardiac Health Score Visualisation reflecting patient adherence  10-year Risk Trajectory Charts  Trend Charts & Peer Ranking **Provider Dashboard (via the care-provider app and cloud platform)** Patient Data Aggregation  Health Report & Goal Review  Intervention Management  Follow-Up Workflows  Analytics & Reporting |
| Daniels et al. (2023), mHealth app | Older adults and the study team/ researchers | **Older adults:** Progress screen with exercise charts and feedback snippets for self-monitoring  Community Calendar  Personalised “home” view summarising baseline activity, motivation profile, and goals on entry **Backend Analytics Dashboard (for researchers)** Internal research-facing dashboard surfacing usage logs, notification metrics, and aggregate progress data |
| Davies et al. (2024), EMBED Car | **Professionals (**senior nurse or care-home manager, community nursing leads, GPs in primary care) | **Currently implemented:** Patient/Resident roster view with latest IPOS-Dem scores Colour-coded alert indicators Trend-tracking displays of symptom-and-concern scores Filtering and prioritisation controls (by alert status, date of last assessment, etc.)  **Proposed or under refinement for upcoming trials:** Action-logging functionality to record responses/actions taken against alerts (e.g. task assignments, follow-up notes) |
| Doyle et al. (2021), ProACT platform with CareApp | Older adults, informal and formal carers, and a clinical triage dashboard for dedicated triage nurses monitoring large cohorts | **CareApp (for older adults)** “Flower” Dashboard with colour-coded petals for each tracked parameter,  Time‐series charts of vitals and well-being metrics, with simple zoom controls “My Goals” Dashboard with weekly progress bars for activity goals, plus recommendations from the Goal Recommender **CareApp ( for carers & healthcare professionals)** Read-only views of the person’s flower status, trend charts, and alert notifications (permission‐based sharing) **Clinical Triage Dashboard (SIMS-Triage)** Prioritised alert list (red/yellow/green)  Status tags (New/Under Review/Resolved)  One-click drill-downs into individual trend data |
| Hawley-Hague et al. (2020), "My Activity Programme" and "Motivate Me" apps | Patients and healthcare professionals | **Motivate Me (for healthcare professionals):** “Home” view listing each patient’s programmed exercises, self-reported completion status, and any messages sent. **My Activity Programme (for patients):** Main screen shows daily exercises, enables logging each one, and surfaces incoming motivational pop-ups. Functions as a personal “dashboard” by always displaying the current program and any outstanding tasks. |
| Hilberger et al. (2025), LETHE App and CTMS (clinical trial management system) | Older adults (participant app) and healthcare professionals (CTMS) | Centralised clinician dashboard showing aggregated participant data  Longitudinal tracking of lifestyle, cognitive, and activity metrics  Alerts and overview panels to support trial monitoring and intervention tailoring  Integrated data visualisation linking app‑reported and clinical data |
| Hoffman et al. (2020), Web-based long-term care decision aid | Older adults,  family caregivers,  Ageing Resource Centre staff and healthcare advisors | **Side-by-side comparison chart**  Automatically generated, personalised "My Decision Summary" page printout summarising user inputs (knowledge, preferences, gaps), integrating input data into an actionable format **Interactive input fields** |
| Nambisan et al. (2022), myHESTIA (Comprehensive Digital Self-Care Support System) | Older adults, Informal caregivers and geriatric healthcare professionals | Tabular reports of raw daily tracker entries  Simple line/bar charts displaying trends over time  Gamified “play with my data” tool for exploring statistical correlations between conditions Auto-customised dashboard elements based on the user’s selected chronic conditions |
| Sien et al. (2024), Mantra | Older adults with cancer and multimorbidity Shareable summaries for informal caregivers and healthcare providers. | Daily symptom and event tracking  Emoji-based mood tagging  Weekly summaries in both text and graphical formats  Customizable reminders for medications and reporting  Printable and shareable reports for care providers  Autosave and skip logic to reduce reporting burden  Calendar-based navigation for intuitive data entry  Symptom-triggered educational pop-ups with trusted resources |
| Villa-Garcia et al. (2022), Integrated Care Platform | Chiefly for social workers and, by extension, other care-team members | **Summary overview** of the person’s clinical, functional, cognitive, mental-health and social needs **Care-plan snapshot** showing current objectives, upcoming activities or visits (an agenda view). **Visual alerts** for key issues (e.g. unmet goals or flagged assessment items). **Care-team roster,** clarifying roles/permissions and who’s responsible for each element of the plan. |

Table 2 Details on the dashboard features of the interventions reported in studies
