# Supplementary material for: Human-centred design of digital health dashboards in care of older adults: a scoping review
Source: BMJ Open. 2026 Jul 17;16(7):e113525. doi: 10.1136/bmjopen-2025-113525 (PMC13384139; doi:10.1136/bmjopen-2025-113525)
Supplement: online supplemental table 3 [file bmjopen-16-7-s007.docx]

| Study | Discover | Define | Develop | Deliver |
| --- | --- | --- | --- | --- |
| Abujarad et al. (2021) | Literature review | Developed an initial concept | Iterative prototyping - focus groups followed by a paper survey | Usability testing - Structured 121 walkthrough of the tool, audio/video recording of the think-aloud sessions |
|  | Stakeholders focus groups |  |  | qualitative and quantitative data collection through survey |
| Afolabi et al. (2025) | Stakeholder consultations to explore medication management needs | Synthesis of priorities through committee discussion | Co‑creation of a MVP through iterative feedback | Not reported (future evaluation planned) |
| Bao et al. (2025) | Literature review | Not reported | Iterative prototyping using 3 participatory design cycles | 4-week Pre‑post intervention evaluation with usability, acceptance, and sensor data |
| Cella et al. (2021) | Brainstorming | Identified challenges, | Developed collaborative dashboard mock-ups | Pilot-Tested Dashboard Usability |
|  | Evaluated current workflows | Suggested solutions, | Built a design consensus | Integrated the dashboard with Electronic Health Record |
|  |  | Established design priorities for the dashboard | Parallel focus groups for feedback on design and content appropriateness and desirability |  |
| Chaudhry et al. (2022) | Baseline established using Short Form 12 Item version 2 (SF12v2) Health Survey (incomplete responses imputed by STATA) and Older Persons Quality of Life Survey (OPQoL) | Not reported | Interactive prototyping - using an HCD approach involving the target population and care providers over a period of 5 years | Feasibility testing - 24-week trial, tablets were provided, followed by an updated SF12V2 and OPQoL survey, compared with baseline using the Wilcoxon rank tests |
|  |  |  | 12 technology workshops followed by semi-structured interviews-analysed by an open coding approach | MS Excel was used to generate simple descriptive statistics from application usage logs. |
| Chen et al. (2024)(29) | End user needs analysis (preliminary work reported elsewhere) | Defined three components: patient app, care provider app, and cloud platform. | Iterative design - Guided by user-centred design principles with a multidisciplinary team. | Two-stage usability testing - Stage I: Task analysis (8 tasks) and usability evaluation (modified Health-ITUES (Health Information Technology Usability Evaluation Scale)) with 88 hospitalized Chronic Heart Disease (CHD) patients; |
|  |  |  | Key functions and modules drafted via mind mapping software | Stage II: Usability evaluation (modified Health-ITUES) with 61 CHD individuals who used the app for at least six months |
|  | Literature review |  | Prototyping - Alpha and beta versions developed and tested |  |
| Daniels et al. (2023)(5) | Interviews - Semi-structured, 1-1, lasting between 45-80 minutes | Data described, summarised and interpreted using thematic analysis | Iterative prototyping- a co-creative process involving older adults and experts using multiple mock-ups | Prototype testing - pre-defined protocol of specified tasks performed by each participant using a think-aloud protocol, closely observed by researchers |
|  |  |  |  | Survey consisting of 22 questions, including the SUS (System Usability Scale) and UEQ (User Experience Questionnaire), combined with 14 specific questions |
| Davies et al. (2024)(24) | Co-design process: Involved 20 diverse constituents in brainstorming | Synthesised evidence using a matrix through approaches adapted from framework analysis | Collaborative dashboard prototyping during the co-design process | Usability pilot-test: Conducted to optimise the dashboard's integration with the EHR |
|  | Literature review |  |  | Single-group, pre-post-test study design: Evaluated the dashboard's use among 157 participants with advanced cancer or chronic kidney disease (CKD) from June 2020 to January 2022 (~18 months) |
| Doyle et al. (2021) | Formative studies - interviews, focus groups and co-design sessions | Not reported | Interface redesigns following usability sessions, evaluated by semantic thematic analysis | Prototype testing - 12-month evaluation of user adoption, technology satisfaction, and experiences, to inform interface updates |
| Hawley-Hague et al. (2020)(30) | Informal consultations | Synthesised findings - drafted an initial mock-up in the form of wireframes | Iterative prototyping – Patient Public Involvement workshops implementing notetaking and participant observations, patient interviews in home settings | Prototype testing - 3-week-long usability and acceptability trial |
|  | Literature review |  | mapping of Behaviour Change Techniques to Behaviour Change Taxonomy |  |
| Hilberger et al. (2025) | LETHE app: workshops with stakeholders | User persona creation | Wireframes based on Crazy 8 method | Usability testing – Think aloud method |
|  |  |  | Prototyping in Figma |  |
|  | LETHE CTMS: Literature review | Initial mock-up creation based on literature | Defined CTMS requirements with health professionals | Technical implementation |
|  |  |  | Redesign of mock-ups |  |
| Hoffman et al. (2020)(31) | Literature review - scoping review conducted by researchers | Determined priority design elements | Iterative prototyping -of a Paper prototype through storyboarding | Prototype testing - initially with staff and volunteers before officially launching on the Internet |
|  | Evaluation of existing resources |  |  | Think-aloud testing using Morae software, followed by semi-structured interviews |
| Nambisan et al. (2023)(3) | Interviews - Open-ended | Findings were integrated to inform prototype development | Iterative prototype development- following guidelines from the National Association for Specific Health Conditions | Prototype testing and usability evaluation- 4 weeks of testing followed by surveys and questionnaires |
|  | Literature review |  | User interviews, outcomes measured through PROMIS | Average user experience calculated across four directions (pragmatic, hedonic, sociability, usability) for each individual and each direction |
|  |  |  |  | Log data was used to track participant engagement during and post-study |
| Sien et al. (2024) (33) | Literature review | Development of a conceptual model based on Grey’s Self-Management Theory | Low-fidelity prototyping using Axure and Sketch | Usability testing (remote via Zoom and in-person) with older adults and caregivers using the System Usability Scale (SUS) |
|  | Brainwriting sessions with older adults | Mapping user needs to app features | Medium-fidelity prototyping using Figma | Task completion tracking |
|  | Persona development | Prioritisation of tasks and functions | Iterative design incorporating human-computer interaction principles using RITE (Rapid Iterative Testing and Evaluation) method | Post evaluation qualitative interviews |
| Villa-García et al. (2022)(32) | Analysis of end user needs, home care model and usual practice- Focus groups (older adults and formal caregivers), interviews (Informal caregivers), Delphi consensus (health and social care professionals, integrated carer and technology experts) | Findings translated into a set of use cases indicating functional and non-functional requirements | Co-creation workshops among the driving team to analyse formulated requirements, and align those with clinical workflows, technical complexity, relevance risks and obstacles in complexity | Prototype usability and feasibility testing -by 3 people from each stakeholder group, individually in a laboratory situation, performing predefined tasks, any suggestions or problems were documented |
|  | Literature review |  | Iterative prototyping with the health and social care group, evaluations performed at each iteration | Usability testing - version 1 of the platform in routine care by social workers who provided real-time feedback and older adults |
|  |  |  | Weekly meetings of software developers and designers to evaluate progress, refine concepts and make decisions to generate an MVP |  |
| HCD = Human‑Centred Design, UCD = User‑Centred Design, MVP = Minimum Viable Product, SUS = System Usability Scale, UEQ = User Experience Questionnaire, SF‑12v2 = Short Form 12‑Item Health Survey (Version 2), OPQoL = Older Persons’ Quality of Life questionnaire, STATA = Statistical software package, MS = Microsoft, CHD = Coronary Heart Disease, Health‑ITUES = Health Information Technology Usability Evaluation Scale, EHR = Electronic Health Record, CTMS = Clinical Trial Management System, RITE = Rapid Iterative Testing and Evaluation, PROMIS = Patient‑Reported Outcomes Measurement Information System, PPI = Patient and Public Involvement | | | | |

*Table 3 HCD methods mapped to the double diamond framework*
