# Supplementary material for: Human-centred design of digital health dashboards in care of older adults: a scoping review
Source: BMJ Open. 2026 Jul 17;16(7):e113525. doi: 10.1136/bmjopen-2025-113525 (PMC13384139; doi:10.1136/bmjopen-2025-113525)
Supplement: Supplementary file 8 [file bmjopen-2025-113525.reviewer_comments.pdf]

PEER REVIEW HISTORY

BMJ Open publishes all reviews undertaken for accepted manuscripts. Reviewers are asked to complete a checklist review form and are provided with free text boxes to elaborate on their assessment. These free text comments are reproduced below.

ARTICLE DETAILS

Title (Provisional)

Human Centred Design of digital health Dashboards in care of Older Adults: A Scoping Review

Authors

Arslan, Syeda Taqya Amna; O'Connor, Siobhan; Karki, Bibhusha; French, Chloe; Stanmore, Emma

---

VERSION 1 - REVIEW

**Reviewer**	**1**
**Name**	**Corte-Real, Ana**
**Affiliation**	**University of Coimbra, Faculty of Medicine**
**Date**	**03-Feb-2026**
**COI**	**None**

The manuscript addresses a timely and relevant topic. However, in line with the BMJ Open editorial criteria regarding methodological transparency, reproducibility, analytical rigour and policy and practice relevance, several substantial issues must be addressed before it can be considered further.

With regard to the transparency and reproducibility of the research strategy, the authors must justify their selection in relation to the study objective and the chosen basis. The choice of time interval lacks explicit justification. What is the scientific and contextual relevance of this selection? Setting the upper limit at 2024 is particularly restrictive and may exclude recent evidence in a rapidly evolving field. It is therefore recommended that this limit is either robustly justified or reconsidered. While the review covers the pandemic period, the impact of the pandemic is not discussed. The authors should clarify whether and how the pandemic influenced the designs of the included studies and the differences between studies conducted before, during and after the pandemic. This analysis is particularly relevant for studies in digital health and information systems.

While the assessment of study quality using the MMAT and JBI checklists is methodologically relevant, the authors should: This should be presented as a section of the main manuscript

and not just in an appendix. They should also justify their choice of assessment tools and clarify how the quality assessment influenced the synthesis and interpretation of the results.

With regard to data extraction and synthesisdetails are lacking on the following: (i) procedures to ensure consistency between reviewers, (ii) resolution of discrepancies, and (iii) the link between extracted data and presented results.

The article should provide greater methodological detail. It is unclear whether the social and economic determinants of health were considered. The authors should clarify whether factors such as socioeconomic inequalities, access to technology, digital literacy, and cultural context were analysed. If not, they should explicitly justify their exclusion and acknowledge this as a limitation. This dimension is crucial for the applicability of the results in real contexts.

The role of stakeholders is not sufficiently developed. The authors should specify: Who were considered stakeholders, at what stages of the process were they involved, and what impact did their involvement have on the results? This aspect is particularly relevant for studies with implications for practice, system design, and health policies.

**Reviewer**	**2**
**Name**	**Okpalauwaekwe, Udoka**
**Affiliation**	**University of Saskatchewan, Medicine**
**Date**	**03-Mar-2026**
**COI**	**None**

Thanks for the opportunity to review your work. Overall, a strong scoping review with clear structure, good methodology and meaningful synthesis. Below are focused comments highlighting areas that may benefit from clarification or tightening:

1)There is an inconsistency in your search dates. The abstract reports searches up to February 24, 2024, while the methods section states January 2012 through December 2024.

2) The operational definition of “dashboard” is broadened to include tools even if the term is not explicitly used. While this is reasonable, the inclusion criteria do not clearly specify what minimum features qualified an intervention as a dashboard. Add one or two explicit operational markers (e.g., visualised summary displays, interactive data visualisation, longitudinal tracking interface) to clarify and strengthen this section.

3) Human-centred design (HCD) and user-centred design (UCD) appear to be treated interchangeably. Since these are conceptually related but not identical, a brief clarification in the methods explaining whether and why they were grouped together would pre-empt conceptual critiques.

4)The “Define” phase in the Double Diamond framework is identified as underdeveloped across studies. This is an important conceptual insight, but your discussion does not fully explore its implications. Why?

5) The limited use of dashboard analytics and usage logs is repeatedly noted and is one of the strongest contributions of the review. However, your discussion remains largely descriptive. You can link this to the absence of analytics to limitations in iterative refinement, implementation science, and learning health system development.

6) All included studies are described as high quality, yet this is a scoping review. Since scoping reviews typically do not exclude studies based on quality, it would be helpful to clarify that appraisal was conducted descriptively and did not influence inclusion or synthesis weighting.

7)Your discussion contains some repetition, particularly regarding post-2020 publication trends, geographic concentration in Europe/North America, and inconsistent framework use.

8) You state that HCD led to improved usability, self-management, and emotional engagement. Given that most included studies were feasibility or usability studies rather than effectiveness trials, slightly tempering this language (e.g., “suggest potential improvements”) would better align claims with the evidence base.

9) The comparison to public health dashboard literature is useful but slightly abrupt. Adding one bridging sentence explaining why contrasting clinician- or policy-facing dashboards with older adult–focused dashboards matters would improve narrative flow.

10) There are a few minor typographical and formatting issues that should be corrected before final submission (e.g., duplicated citation brackets, minor spelling errors such as “clincal,” and a missing period in the conclusion section).

VERSION 1 - AUTHOR RESPONSE

Reviewer 1

Comment: With regard to the transparency and reproducibility of the research strategy, the authors must justify their selection…

Response: We have conducted an additional search until 30th March 2026 to reflect the most updated research

Comment: While the review covers the pandemic period, the impact of the pandemic is not discussed…

Response: We have added a dedicated paragraph in the Results section addressing how the COVID-19 pandemic may have influenced study designs, digital adoption, and implementation contexts. Addition has also been made in the Discussion section.

Comment: While the assessment of study quality using the MMAT and JBI checklists is methodologically relevant…

Response: In line with scoping review guidelines, quality appraisal has only been discussed descriptively

Comment: With regard to data extraction and synthesisdetails are lacking on the following…
Response: Study selection section has been updated

Comment: The article should provide greater methodological detail…
Response: Where reported, data was extracted on factors related to access to technology, digital literacy, and contextual or cultural influences on participation and use. Data extraction and Discussion sections have been subsequently updated.

Comment: The role of stakeholders is not sufficiently developed…
Response: Updated in the Results section under study characteristics as well as in discussion section.

Reviewer 2

Comment: There is an inconsistency in your search dates…
Response: This inconsistency has been corrected, and all sections now report aligned search dates.

Comment: The operational definition of "dashboard" is broadened to include tools even if the term is not explicitly used…
Response: We have strengthened the inclusion criteria by adding explicit operational markers (e.g., visualised summary displays, interactive data visualisation, and/or longitudinal tracking features) to clarify what qualified as a dashboard with the Methods Section under the inclusion criteria

Comment: Human-centred design (HCD) and user-centred design (UCD) appear to be treated interchangeably…
Response: A brief clarification has been added to the Methods section explaining why HCD and UCD were grouped, while acknowledging their conceptual distinctions within the Inclusion criteria

Comment: The "Define" phase in the Double Diamond framework is identified as underdeveloped across studies…
Response: Discussion section has been updated.

Comment: The limited use of dashboard analytics and usage logs is repeatedly noted and is one of the strongest contributions of the review…
Response: The Discussion now explicitly links the absence of analytics and usage logs to limitations in iterative refinement, implementation science, and learning health system development.

Comment: All included studies are described as high quality, yet this is a scoping review….
Response: We clarify that appraisal was descriptive only and did not affect inclusion or synthesis weighting.

Comment: Your discussion contains some repetition…
Response: We have reduced repetition and streamlined overlapping sections in the Discussion.

Comment: You state that HCD led to improved usability, self-management, and emotional engagement…
Response: We have revised language throughout to ensure claims are appropriately cautious and aligned with the predominantly feasibility-focused nature of the included studies.

Comment: The comparison to public health dashboard literature is useful but slightly abrupt…
Response: A bridging sentence has been added to clearly explain the relevance of this comparison.

Comment: There are a few minor typographical and formatting issues that should be corrected before final submission ...

Response: All identified typographical and formatting issues have been corrected.

We sincerely thank the Editor and Reviewers again for their thoughtful and constructive feedback, which has significantly strengthened the manuscript. We hope the revisions adequately address all concerns, and we look forward to your further consideration.

VERSION 2 - REVIEW

**Reviewer**	**1**
**Name**	**Corte-Real, Ana**
**Affiliation**	**University of Coimbra, Faculty of Medicine**
**Date**	**16-Jun-2026**
**COI**	

The authors have responded comprehensively to the points raised in my initial review. They have extended and clarified the search strategy, explicitly addressed the impact of the COVID-19 pandemic, and provided greater detail on data extraction, quality appraisal and the use of HCD frameworks. The role of stakeholders is now more clearly described, and contextual factors such as access to technology and digital literacy are acknowledged and discussed as limitations. Overall, I consider that the major concerns from the previous round have been adequately addressed.

**Reviewer**	**2**
**Name**	**Okpalauwaekwe, Udoka**
**Affiliation**	**University of Saskatchewan, Medicine**
**Date**	**01-Jun-2026**
**COI**	

Well done with the revisions and responses to reviewer comments. I appreciate the improved wuality of your work.
